# Ostrich eggshell beads reveal 50,000-year-old social network in Africa

**DOI:** 10.1038/s41586-021-04227-2

**Published:** 2021-12-20

**Authors:** Jennifer M. Miller, Yiming V. Wang

**Affiliations:** 1 Department of Archaeology, Max Planck Institute for the Science of Human History, Jena, Germany; 2 Department of Anthropology, University of Alberta, Edmonton, Alberta Canada

**Keywords:** Palaeoclimate, Archaeology

## Abstract

Humans evolved in a patchwork of semi-connected populations across Africa^1,2^; understanding when and how these groups connected is critical to interpreting our present-day biological and cultural diversity. Genetic analyses reveal that eastern and southern African lineages diverged sometime in the Pleistocene epoch, approximately 350–70 thousand years ago (ka)^3,4^; however, little is known about the exact timing of these interactions, the cultural context of these exchanges or the mechanisms that drove their separation. Here we compare ostrich eggshell bead variations between eastern and southern Africa to explore population dynamics over the past 50,000 years. We found that ostrich eggshell bead technology probably originated in eastern Africa and spread southward approximately 50–33 ka via a regional network. This connection breaks down approximately 33 ka, with populations remaining isolated until herders entered southern Africa after 2 ka. The timing of this disconnection broadly corresponds with the southward shift of the Intertropical Convergence Zone, which caused periodic flooding of the Zambezi River catchment (an area that connects eastern and southern Africa). This suggests that climate exerted some influence in shaping human social contact. Our study implies a later regional divergence than predicted by genetic analyses, identifies an approximately 3,000-kilometre stylistic connection and offers important new insights into the social dimension of ancient interactions.

## Abstract (web summary)

By tracing the changing size of ostrich eggshell beads, climate is shown to have an important role in influencing when and where regional African populations interacted.

## Main

Unresolved questions in human evolution concern the ancient distribution and diversification of our species (*Homo sapiens*) across Africa^2,5^. The metapopulation model suggests that anatomical modernity and behavioural complexity arose within a pan-African patchwork of populations who experienced pulses of connection and isolation^6^, possibly in response to environmental circumstances^1,7^. Research into these shifting connections is increasingly derived from DNA and ancient DNA analyses, which reveal that present-day African hunter–gatherer populations diverged into regional lineages sometime in the Pleistocene, including a deep division between southern and eastern groups approximately 350–70 ka^3,4,8^. Although ancient DNA is a powerful tool for acquiring information about biological exchange, it is unable to address the cultural context of ancient interactions. Many questions about these ancient interactions remain, such as where and when did ancient populations connect, what social exchanges took place and what mechanisms provoked their eventual isolation.

Beginning in Marine Isotope Stage 3 (approximately 57 ka), African populations underwent substantial social reorganization^9–11^. Numerous advancements appear around this time, but an important new feature is the manufacture of beads^12^ (Supplementary Discussion 1). The systematic production of beads is a considerable labour investment, and signals the increasing scale and importance of social interactions in Marine Isotope Stage 3 (ref. ^13^), perhaps relating to the growing population size and social systems evident around this time^11^. These societal reforms signal that the African Late Pleistocene is a crucial period for understanding the development of complex social networks.

Ostrich eggshell (OES) beads are the oldest fully manufactured beads and could be key to revealing Late Pleistocene social dynamics in Africa. They emerged in eastern Africa by 52 ka^12^, in southern Africa by 42 ka^14^ and are still produced in some areas today. Modern ethnographic research in Africa indicates that a finished piece of OES beadwork (for example, a beaded skirt) carries symbolic meaning^15^. However, individual beads can also preserve social information, as every step in their production is a deliberate choice that intensifies morphological differences^16^ (Supplementary Discussion 2). These manufacturing decisions are cultural norms that are commonly shared between neighbouring groups, while long distances reduce transmission opportunities leading to cultural variation or drift^17–20^. Therefore, the characteristics of OES beads can be used as a means to reconstruct population interaction. Previous studies linked the introduction of herding into southern Africa (approximately 2 ka) with the appearance of larger-diameter OES beads^21,22^, indicating possible connections with eastern African populations, as supported by archaeological and genetic evidence^4,21,23^. Some recent studies have reported stylistic variation within Late Pleistocene sites^24–27^; however, to our knowledge, there has been no attempt to use similar variation to explore population contact in the Pleistocene.

Episodes of population connection and isolation have been linked with environmental shifts^1,2^, and over the past 50,000 years (kyr), climatic events have triggered temperature fluctuations and hydroclimatic reorganization in Africa^28–30^. These shifts could have fragmented habitable areas, in turn affecting where and when regional populations could interact. Therefore, it is critical to explore how intergroup connectivity may correspond with climatic and environmental changes in the Late Pleistocene.

**Fig. 1:**
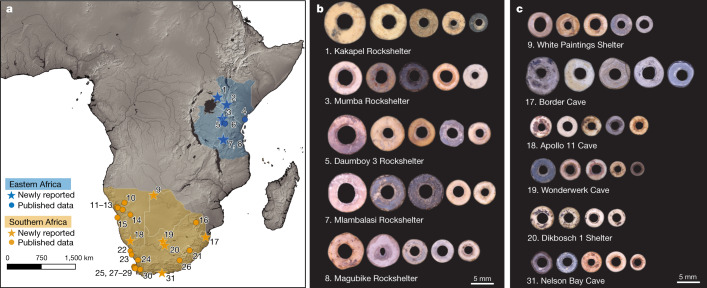
Locations of sites included in this study and palaeoclimate records. Base map modified from Natural Earth. **a**, Kakapel Rockshelter (1); Enkapune ya Muto (2); Mumba Rockshelter (3); Panga ya Saidi (4); Daumboy 3 Rockshelter (5); Kisese II Rockshelter (6); Mlambalasi Rockshelter (7); Magubike Rockshelter (8); White Paintings Shelter (9); Geduld (10); Lower Numas Cave (11); Lower Orabes Shelter (12); Leopard Cave (13); Eros (14); Wortel (15); Bushman Rockshelter (16); Border Cave (17); Apollo 11 Cave (18); Wonderwerk Cave (19); Dikbosch 1 Shelter (20); Sehonghong (21); SK2001.026 (22); Rooiwal Hollow/Midden (23); Varsche Rivier 003 (24); Paternoster (25); Grassridge Shelter (26); Witklip (27); Kasteelberg A + B (28); Geelbek Dunes (29); Voelvlei (30); Nelson Bay Cave (31). **b**, Representative OES beads from sites in eastern Africa. **c**, Representative OES beads from sites in southern Africa.

In this study, we analysed OES bead characteristics from the past 50 kyr in search of patterns that reveal population connections, and their association with hydroclimate shifts in Africa. We compiled data from 31 sites in eastern (22.5–40° E, 9° N to 9° S) and southern Africa (8–35° E, 20–35° S), totalling 1,516 individual beads (Fig. 1, Supplementary Table 1), with 1,238 of these being fully reported for the first time. We recorded three metric variables wherever possible (bead diameter, aperture diameter and shell thickness). Our database comprises securely dated Pleistocene sites with available data, and well-dated sequences in each region, with age estimates drawn from direct radiocarbon dates, dated archaeological layers or bracketing layers. To understand the potential effects of climate on these patterns, we divided the past 50–2 kyr into four periods based on major glacial and interglacial shifts (Supplementary Discussion 3): phase I: 50–33 ka (Marine Isotope Stage 3 to the reinvigoration of ice-sheet growth); phase II: 33–19 ka (ice-sheet growth to the end of Last Glacial Maximum); phase III: 19–11.6 ka (last deglaciation); and phase IV: 11.6–2 ka (Early Holocene epoch to before the spread of herding into southern Africa). Phase V (2 ka to present) marks the previously identified shift in bead sizes that emerges as herding spreads into southern Africa. We expect to see population connections indicated by similar bead characteristics, and that periods of isolation may parallel climatic shifts.

## Regional and chronological bead metrics

**Fig. 2:**
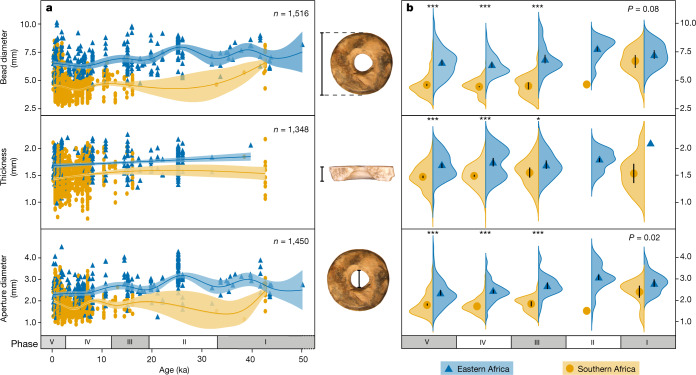
OES bead diameter, thickness and aperture diameter distribution through the past 50 kyr in eastern and southern Africa. **a**, Generalized additive model plots to show bead characteristic evolutionary trajectories. The mean (curved lines) and 95% confidence interval are shown for each parameter. **b**, Split violin plots of bead parameters. The violins represent the kernel density of the frequency distribution, and the points are presented as mean values ± one standard error. Statistical results are shown in Supplementary Tables 5–7, 11. The asterisks denote the significance of region differences (*P < 0.01 and ***P < 0.001). When no significance between two regions was found, *P* values are presented.

Our results reveal that eastern and southern African OES beads take unique stylistic trajectories through time (Fig. 2a). Phases and regions are both important factors driving the variation in OES bead characteristics (Pillai’s trace = 0.60, *F*_3,1319_ = 664.8, *P* < 0.001 for region and Pillai’s trace = 0. 18, *F*_12,3963_ = 21.34, *P* < 0.001 for phase), although interaction between phases and regions do not appear to significantly influence OES bead characteristics (Pillai’s trace = 0.02, *F*_9,3963_ = 2.22, *P* = 0.02; Supplementary Table 2).

**Fig. 3:**
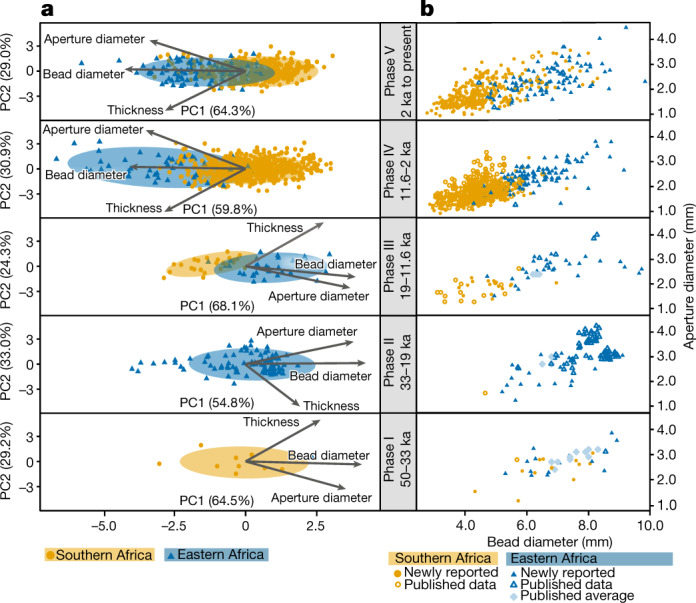
Comparison of bead characteristics between eastern and southern Africa during the past 50 kyr. **a**, Principal component analyses (PCA) of OES bead metric parameters for phases III–V. Diameter, aperture diameter and thickness account for more than 89% of the variation, separating eastern and southern Africa into distinct groups. PCA clustering for two regions for phase II and phase I is not possible due to insufficient data. **b**, Paired diameter and aperture diameter for each phase. Newly reported data: collected by authors, reported as individual beads; published data: drawn from published metrics, reported as individual beads; average data: drawn from published metrics, reported as averaged values.

In eastern Africa, the range of bead and aperture diameters remain consistent over 50 kyr, with only minor fluctuations. Eastern beads average 6.9 ± 1.2 mm in diameter and 2.6 ± 0.6 mm in aperture diameter (Fig. 2a), with a wide range of variation. By contrast, southern bead characteristics have changed through time, with larger bead and aperture diameters in phase I (50–33 ka) and significantly smaller characteristics in the younger phases (Pillai’s trace = 0.113, *F*_9,3147_ = 13.4, *P* < 0.001; Fig. 2a, Supplementary Table 3). While southern beads virtually disappear from the archaeological record in phase II (33–19 ka), they re-emerged around the onset of deglaciation (approximately 19 ka) with consistently smaller sizes. From phases III–V (19 ka to present), southern bead diameters and aperture diameters are smaller with narrower ranges (4.5 ± 0.9 mm and 1.8 ± 0.4 mm, respectively) than their eastern counterparts. They remained in this consistently smaller style until after 2 ka when larger bead characteristics, associated with the movement of pastoral communities, appeared in southern Africa (multivariate analysis of variance (MANOVA) Pillai’s trace = 0.004, *F*_3,700_ = 1.05, *P* = 0.371; Supplementary Table 4) (Figs. 2b, 3).

We found distinct regional clusters with varying degrees of overlap throughout phases III–V (19 ka to present) using principal component analysis for specimens with all three metric parameters (*n* = 1,333) (Fig. 3a). PC1 and PC2 explain 92%, 91% and 93% of variations between southern Africa and eastern Africa for phase III (19–11.6 ka), phase IV (11.6–2 ka) and phase V (2 ka to present), respectively (Fig. 3a). The univariate analysis of variance (ANOVA) performed on the MANOVA outputs showed that all three parameters have a role in driving the regional differences in phases III–V (ANOVA *P* < 0.001 for all tests; Supplementary Tables 5–7). We further explored these regional differences using the two most commonly reported variables (bead diameter and aperture diameter), which slightly increased sample size to 1,445 beads (Fig. 3b). Our MANOVA results using only these two variables confirmed that bead characteristics are significantly different between the two regions during phases III–V (19 ka to present) (Fig. 3b, Supplementary Tables 8–10). Compared with the more distinct regional bead clusters in phase III (19–11.6 ka) and phase IV (11.6–2 ka), the beads in phase V (2 ka to present) show increased overlap between eastern and southern Africa. Despite this overlap, most southern beads in phase V (2 ka to present) remain smaller, consistent with phase III (19–11.6 ka) and phase IV (11.6–2 ka) (Figs. 2b, 3b).

Bead characteristics in phase I are nearly identical for eastern and southern Africa (Pillai’s trace = 0.15, *F*_2,36_ = 3.2, *P* = 0.052; Figs. 2b, 3b, Supplementary Table 11), with similarities driven by bead diameter and aperture diameter (ANOVA *P* = 0.08 and 0.02, respectively; Supplementary Table 11). The average OES bead diameters in southern Africa are larger in phase I (6.7 mm) than those in other time periods by more than 2 mm, making them more similar to sizes in eastern Africa (average diameters of more than 6.9 mm) (Fig. 3b). The majority of southern beads (12 out of 14) derive from a single site—Border Cave—which has a wide range of diameters (4.3–8.1 mm). The remaining beads are one each from VR003 and White Paintings Shelter. Both sites are located significantly further west, but each bead is 5.7 mm in diameter, which falls within the range of diameters from Border Cave.

Shell thickness is not a stylistic trait, but instead may reflect a complex relationship between environment and ostrich. Both regions maintain consistent shell thickness over the entire 50 kyr period, with eastern African shells averaging 1.7 ± 0.2 mm, and southern shells averaging 1.5 ± 0.2 mm (Fig. 2b). This appears to contradict previous suggestions that shell thickness varies in response to temperature and aridity^31^. While thickness does not vary within each region through time, it is significantly different between the two regions (*P* < 0.004 for phases III–V; Supplementary Tables 5–7), and may represent different ostrich sub-species^32,33^. The thinner southern African shell may have encouraged the production of smaller beads, and future studies should test this hypothesis, although this would not account for larger beads in southern African phase I (50–33 ka) and phase V (2 ka to present).

## Discussion

### Stylistic connection at 50–33 ka

**Fig. 4:**
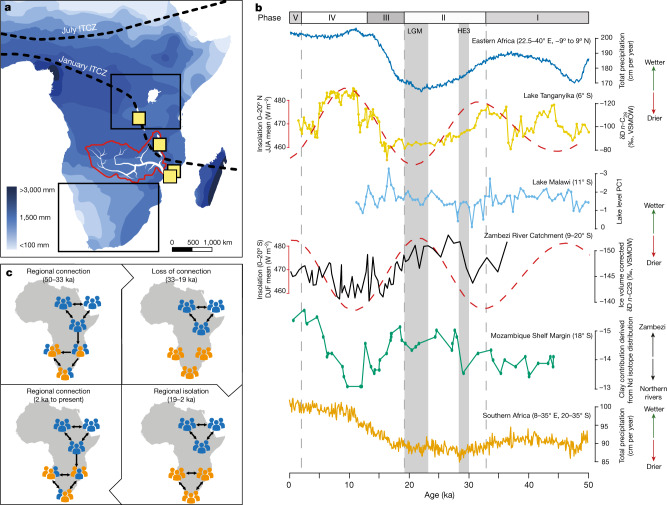
Bead-derived social connection and comparison climate proxies from eastern and southern Africa. **a**, Annual mean rainfall map of Africa (map modified from Wikimedia Commons under the CC BY-SA 4.0 licence), showing the position of palaeoclimate data (yellow squares) used in **c**: Lake Tanganyika^29^; Lake Malawi^46^; Core GIK16160-3 (ref. ^30^); Core 64PE304-80 (ref. ^43^); Zambezi River catchment area (red) and major tributaries (white). The two black boxes indicate the modelled climate data area for eastern Africa (22.5–40° E, −9 to 9° N) and southern Africa (8–35° E, 20–35° S) from the LOVECLIM transient climate model^35^. The black dashed lines indicate the location of present-day Intertropical Convergence Zone (ITCZ) in January and July. **b**, Latitudinal north to south sequence of climate data series (from top to bottom): total precipitation for eastern Africa (22.5–40° E, −9 to 9° N) derived from the LOVECLIM transient climate model^35^; moisture availability derived from the δD of leaf wax record from Lake Tanganyika^29^ is overlaid with the June–July–August (JJA) mean insolation at 0–20° N (red dashed line); lake level is derived from PCA analyses (PC1) in Lake Malawi; moisture availability derived from the δD of leaf wax record (ice volume corrected) from the Zambezi River catchment area^47^ overlaid with the December–January–February (DJF) mean insolation at 0–20° S; the neodymium (Nd) isotope signature of lithogenic fractions in marine sediments from the Mozambique Shelf margin^43^; the total precipitation for southern Africa (8–35° E, 20–35° S) derived from the LOVECLIM transient climate model^35^. HE3, Heinrich Event III; LGM, Last Glacial Maximum; VSMOW, Vienna Standard Mean Ocean Water. **c**, Social connections between eastern and southern Africa, derived through bead styles, over the past 50 kyr.

Despite the substantial distance (more than 3,000 km) between eastern and southern Africa, the available OES beads from phase I (50–33 ka) share stylistic similarities. This is the oldest (and only) time period that the two regions have the same bead diameter range, strongly suggesting some form of socially mediated exchange during this time, marking the furthest Pleistocene stylistic connection ever documented. On the basis of age, site locations and bead characteristics, OES bead technology appears to have originated in eastern Africa. The oldest directly dated eastern beads are approximately 10 kyr older than those from southern Africa^12,14,34^. Most southern beads in this phase come from Border Cave, which is located towards eastern Africa (Fig. 1a); however, none of the three phase I sites from southern Africa have signs of in situ bead production. This apparent spread of beadmaking technology, evident mainly from the traits at Border Cave, corresponds with the relatively wet climatic conditions in eastern Africa during phase I (50–33 ka) (Fig. 4).

### Disconnection and climatic links

The regional network seems to break down sometime in phase II (33–19 ka), raising questions about the influence of climate on social connections (Fig. 4a). By 33 ka, precipitation in eastern Africa decreased (Fig. 4b), modulated by the Indian Winter Monsoon and decreasing sea surface temperature of the Indian Ocean^29^. These drier conditions persisted until approximately 16 ka^29,35^, resulting in the lowest net primary production values over the past 50 kyr, according to climate model simulations (Supplementary Figs. 2, 3). This reduction of net primary production would have altered the distribution of vegetation and fauna on the landscape^7^ (Supplementary Fig. 4), requiring humans to adjust mobility and foraging strategies^36–38^. This in turn could have reorganized the distribution of people on the landscape, depopulating areas, and rendering some previous social networks unsustainable^1,39,40^. The breakdown between phase I and phase II also coincides with the lowest effective population size in Africa predicted by ref. ^41^, and may suggest that shrinking population sizes contributed to regional disconnection. By contrast, the Zambezi River catchment (the large region connecting eastern and southern Africa) became wetter from 30 to 16 ka, according to climate proxy data (Fig. 4b). This enhanced rainfall was due to the southward migration of the Intertropical Convergence Zone to 10–20° S, largely controlled by Heinrich Event III in the North Atlantic where massive iceberg melting occurred approximately 30 ka^42^ (Supplementary Discussion 4). The increased precipitation resulted in periodic flooding of Zambezi River and its tributaries^43^, which could have formed a geographical barrier to connections between eastern and southern Africa (Supplementary Discussion 4, Fig. 4b). The drying trend in eastern Africa and the flooded Zambezi River catchment may have instigated the regional disconnection that appeared by phase II (33–19 ka), suggesting that climate induced behavioural responses could be an important mechanism for driving cultural isolation in the Late Pleistocene (Fig. 4c).

Southern OES beads became rare, even seeming to disappear by 33 ka, and did not re-emerge until after 19 ka (Supplementary Discussion 5). Their absence coincides with the lowest net primary production and the coolest glacial temperatures in southern Africa, which may have limited the population size in the Late Pleistocene. If social group sizes are small, the mass production of standardized beads can be more costly than beneficial (Supplementary Discussion 1). This could explain why OES beadmaking did not become part of the cultural repertoire, even after the technology was introduced in phase I (50–33 ka). When southern beads do re-emerge (approximately 19 ka), they are in an exclusively smaller style. This regionalization of styles reflects a prolonged period of social isolation, and corresponds with a gradual increase in precipitation and temperature in southern Africa (Fig. 4b, Supplementary Fig. 1). Finally, bead styles document another episode of connection after 2 ka when mobile pastoralists enter southern Africa^21^.

### Human resilience and regional adaptions

The distinct trajectories of bead characteristics suggest that populations in each region responded to environmental changes with different social strategies. The eastern bead tradition is continuous, and its characteristics remain steady, regardless of any climatic shifts. This consistency hints at the presence of resilient intraregional social networks that remain intact even throughout 50 kyr of environmental uncertainty. Owing to the overall higher net primary production and carrying capacity, populations in eastern Africa may have sustained larger sizes or more robust social networks as a strategy to mitigate climate change. By contrast, southern African OES bead characteristics vary widely, and bead use was rare from 30 to 19 ka. This may reflect a strategy where populations lived in smaller, disconnected groups, with less need for symbolic behaviour (Supplementary Discussion 1). Other archaeological evidence from this time seems to support this, showing a staggered technological transition in Marine Isotope Stage 2–3 with possible coexisting but culturally unique sub-populations in southern Africa^44,45^. The proliferation of consistently sized beads after 19 ka suggests an increasing reliance on symbolic behaviour after climate conditions improved. These regional differences highlight the flexibility of human social behaviour and illustrate variable strategies for coping with environmental challenges in the Late Pleistocene.

### Perspective

Our research presents a new line of evidence to help to disentangle complex interactions between ancient populations that are difficult to understand through genetic data alone. The stylistic variation of OES beads reveals intermittent connections between eastern and southern African populations over the past 50 kyr, including the oldest regional stylistic connection ever identified. Furthermore, our findings suggest that cultural contact persisted long after the genetic divergence estimate of 70 ka. This raises interesting questions about whether these social connections existed independently from population admixture or coexisted with biological introgression. Future research is warranted to explore these scenarios. In addition, we find it plausible that climatic variability and human behavioural responses affected interregional social networks by conditioning where and when people could meet. Researchers can build on this foundation by incorporating OES bead data from site-based studies to refine the broader regional comparisons (Supplementary Discussion 6).

## Methods

### Data collection

This study reports data from 1,516 OES beads from 31 sites across sub-Saharan Africa (Fig. 1). Of these, 1,238 beads from 11 sites are described, while previously published data includes 290 beads from 21 sites (Supplementary Table 1). We used all relevant data with no statistical methods used to predetermine sample size. We selected completed beads, based on the criteria by Orton^48^: ground to a circular shape, presence of use-wear, or were completed and broken with more than 50% remaining. The majority of the specimens were analysed in-person (*n* = 1,148), under low-power magnification, and photographed with a digital microscope. We recorded three metric variables (bead diameter, aperture diameter and thickness) wherever possible. These are the most frequently reported, standardized characteristics in published literature. Bead colour and shape are less commonly reported and may be more subject to interobserver error, so these and other qualitative variables have been excluded from this study. Bead diameter and aperture diameter both result from cultural behaviour, whereas shell thickness instead may reflect a complex relationship between pore density, environmental aridity^31^ and ostrich sub-species^32^. To obtain diameter values in-person, multiple measurements were taken around the perimeter of the bead using digital calipers. Beads measured from photos with a visible scale were processed in ImageJ to obtain diameter and aperture diameter measurements. As not all beads or apertures are perfectly round, minimum and maximum measurements were used to generate an average, and this value was used in the analysis. Published measurements could not be assessed in the same manner, and in these cases, the reported average diameter of completed beads was used. We only included beads that have estimated age (either by direct dates, dating of an excavation layer or by averaging bracketing dates from surrounding layers). Wherever possible, we calibrated the original radiocarbon age with either Intcal13^49^ or Intcal20^50^. All phase V data were calibrated with Intcal20, and randomly selected ages from other phases were also calibrated with Intcal20^50^. Differences between the two were minor enough that recalibration of the entire dataset was unwarranted. No blinding or randomization were required for this study.

### Statistical analyses

All statistical analyses were performed in R version 4.0.1^51^ with RStudio interface version 1.3.959. We grouped the last 50 kyr into five periods: phase I: 50–33 ka (Marine Isotope Stage 3 to time of renewed ice-sheet growth); phase II: 33–19 ka (the onset of global ice sheet growth to the Last Glacial Maximum); phase III: 19–11.6 ka (last deglaciation); phase IV: 11.6–2 ka (Early Holocene to the spread of herding into southern Africa); and phase V: 2 ka to present (spread of herding into southern Africa to present). Phase I and phase II contain the majority of data points, whereas phase V has the least, and southern Africa only has one point for phase IV.

We grouped the 31 sites into two geographical regions (southern Africa and eastern Africa) instead of examining bead characteristics between sites, for two reasons. First, the number of data points between sites was extremely uneven. The largest dataset (Nelson Bay Cave, South Africa) has *n* = 529, whereas 15 sites have less than six data entries, and seven of these only have one data entry. OES bead data distributed unevenly through time with more OES beads in the later phases than phase I and phase II, probably due to myriad factors. Notably, phase I has 39, phase II has 97 and phases III–V contain 1,380 samples. The sampling difference between regions and time periods is an unavoidable outcome of archaeological data. Second, the differences between sites were negligible compared with the differences between regions. A two-sample *t*-test between southern and eastern Africa shows that for regional difference, the OES diameter was significantly different (two-sample *t*-test, mean diameter = 6.9 mm and 4.5 mm, respectively, *t* = 34.1, d.f. = 510.9, *P* < 0.0001). For example, the mean diameters from the southern African sites of Nelson Bay Cave and Wonderwerk Cave (both of which have more than 350 data points) are 4.4 mm and 4.6 mm, respectively. Whereas the average diameters for eastern African sites Enkapune ya Muto and Mumba Rockshelter (both of which have approximately 80 data points) are 6.7 mm and 6.2 mm. Therefore, we suggest that classifying OES data by region is appropriate. Furthermore, despite eastern Africa having far fewer numbers of beads than southern Africa, they are consistently present throughout all five phases. By contrast, OES beads are largely absent during phase II (33–19 ka), and our dataset only includes one bead from this period.

We applied principal component analysis (PCA; R-package vegan^52^) using multi-dimensional information (bead diameter, aperture diameter and shell thickness) to examine variation in eastern and southern Africa over the past 50 kyr. Out of 1,516 beads, 1,333 had all three parameters available, so these are included in the PCA. A covariance matrix PCA was used to preserve variance because the range and scale of variables are in the same units of measure. We also applied MANOVA to examine the OES bead variation in these two regions through time using the following steps: (1) we conducted a two-way MANOVA to investigate whether the three bead parameters were influenced by region, phases and/or the interactions between region and phases. (2) We applied Pillai’s trace MANOVA to test the null hypothesis that there is no significant difference in OES characteristics between southern Africa and eastern Africa for phases I–III. (3) We applied Pillai’s trace MANOVA to test the null hypothesis that there is no significant difference in OES characteristics through time for each region. We did not conduct any statistical tests for phase II because there are insufficient data available from southern Africa. (4) For phase V, we compared OES beads of two regions based on the diameter and aperture diameter using MANOVA, as thickness records from eastern Africa are incomplete (only one sample has recorded thickness). (5) Finally, we used univariate ANOVA performed on the output from all MANOVA to assess which bead parameters are important for driving the differences in OES sizes in regions and through time.

Although not every specimen has all three parameters recorded, every entry at least has a bead diameter, so analysis by diameter provides the larger dataset. Shell thickness is the least likely to be absent as it cannot be accurately measured from photos and is inconsistently reported. Therefore, to increase sample size and visually demonstrate bead variability, we also created plots that include fewer variables. Specifically, we plotted all reported bead parameters against time, running a generalized additive model for each of these variables in both regions to show understanding of how sizes of these parameters evolve through time. Unless otherwise stated, statistical significance is assessed at *P* < 0.01. All PCA figures were made using ‘ggplot2’ packages^53^.

### Reporting summary

Further information on research design is available in the Nature Research Reporting Summary linked to this paper.

## Online content

Any methods, additional references, Nature Research reporting summaries, source data, extended data, supplementary information, acknowledgements, peer review information; details of author contributions and competing interests; and statements of data and code availability are available at 10.1038/s41586-021-04227-2.

## Supplementary information

Supplementary Information This file contains Supplementary Discussion; Supplementary Figs. 1 – 5; legend for Supplementary Table 1; Supplementary Tables 2–11 and their accompanying legends and Supplementary References.

Reporting Summary

Supplementary Table 1

## Data Availability

All data generated or analysed for this study are included in this published Article (and Supplementary Table 1). All statistical analyses were performed in R version 4.0.151 with RStudio interface version 1.3.959. All PCA figures were made using ‘ggplot2’ version 3.3.5. The data and R code are available from GitHub (https://github.com/alsjmonsoon/Ostrich-egg-shell-bead-data).
