# Supplementary material for: Ostrich eggshell beads reveal 50,000-year-old social network in Africa
Source: Nature. 2021 Dec 20;601(7892):234–9. doi: 10.1038/s41586-021-04227-2 (PMC8755535; doi:10.1038/s41586-021-04227-2)
Supplement: Supplementary file 1 — This file contains Supplementary Discussion; Supplementary Figs. 1 – 5; legend for Supplementary Table 1; Supplementary Tables 2–11 and their accompanying legends and Supplementary References. [file 41586_2021_4227_MOESM1_ESM.pdf]

nature portfolio

https://doi.org/10.1038/s41586-021-04227-2

Supplementary information

Ostrich eggshell beads reveal 50,000-year-old social network in Africa

In the format provided by the authors and unedited

Supplementary Information

Ostrich eggshell beads reveal 50,000-year-old social network in Africa

Jennifer M. Miller*[1,2] and Yiming V. Wang*[1]

[1]Department of Archaeology, Max Planck Institute for the Science of Human History, Kahlaische Str.10, 07745 Jena, Germany
[2]Department of Anthropology, HM Tory Building, University of Alberta, Edmonton, Canada

Supplementary Table 1 (see excel document)
Supplementary Tables 2-11
Supplementary Discussion 1-6
Supplementary Figures 1-5
Supplementary References

Supplementary Table 1 - see excel document

Supplementary Table 2. Two-way Multivariate Analysis of Variance (MANOVA) tests showing bead characteristics (bead diameter, thickness, aperture diameter) influenced by region (Pillai's Trace=0.60, $F_{3,1319}$=664.8, P<<0.001) or phases (Pillai's Trace=0. 18, $F_{12,3963}$=21.34, P<<0.001 for phase), but not their interactions (Pillai's Trace=0.02, $F_{9,3963}$=2.22, P=0.02). Analysis of Variance (ANOVA) were then performed on the output from the MANOVA to assess which bead parameters of ostrich shell bead (OES) sizes were influenced by regions, phases and their interactions. Our ANOVA results show that all parameters were significantly influenced regions and phases (P<0.005).

MANOVA summary

	Df	Pillai	approx F	num Df	den Df	P
Region	1	0.60	664.78	3	1319	<2e-16 ***
Phases	4	0.18	21.34	12	3963	<2e-16 ***
Region:Phases	3	0.02	2.22	9	3963	0.02
Residuals	1321					

ANOVA summary

Response Diameter:

	Df	Sum Sq	Mean Sq	F value	P
Region	1	1419.81	1419.81	1911.53	<2e-16 ***
Phases	4	156.56	39.05	54.67	<2e-16 ***
Region:Phases	3	5.21	1.74	2.43	0.064
Residuals	1321	943.58	0.71		

Response Thickness:

	Df	Sum Sq	Mean Sq	F value	P
Region	1	12.641	12.641	248.398	< 2e-16 ***
Phases	4	0.752	0.187	3.693	0.005 **
Region:Phases	3	0.150	0.050	0.984	0.399
Residuals	1321	67.225	0.051		

Response Aperture Diameter:

	Df	Sum Sq	Mean Sq	F value	P
Region	1	158.883	158.883	743.071	<2e-16 ***
Phases	4	33.652	8.413	39.875	<2e-16 ***
Region:Phases	3	2.782	0.927	4.395	0.004 *
Residuals	1321	278.705	0.211		

Supplementary Table 3. Two-way MANOVA results showing significant differences in OES bead characteristics through time in southern Africa (Pillai's Trace=0.113, $F_{9,3147}$=13.4, P<<0.001). ANOVA performed on the output from the MANOVA show that both diameter and aperture diameter are important bead parameters for driving the differences in OES beads in southern Africa (ANOVA F=33.38, P<<0.001 and F=9.67, P<0.001 for diameter and aperture diameter, respectively). However, thickness has not changed significantly through time in southern Africa (ANOVA F=1.55, P=0.199).

MANOVA summary

	Df	Pillai	approx F	num Df	den Df	P
Phases	3	0.113	13.368	9	3147	< 2.2e-16 ***
Residuals	1049					

ANOVA summary

	Df	Sum Sq	Mean Sq	F value	P
**Response Diameter :**					
Phases	3	60.37	20.123	33.379	< 2.2e-16 ***
Residuals	1049	632.40	0.6029		
**Response Thickness :**					
Phases	3	0.237	0.079	1.554	0.199
Residuals	1049	53.226	0.051		
**Response Aperture Diameter:**					
Phases	3	5.159	1.720	9.666	2.69e-06 ***
Residuals	1049	186.623	0.178		

Supplementary Table 4. Two-way MANOVA results showing no significant difference in bead characteristics between Phases III (19-11.6 ka) and IV (11.6-2 ka) for southern Africa (Pillai's Trace=0.004, $F_{3,686}$=1.05, P=0.371). ANOVA performed on the output from the MANOVA shows that bead parameters are not significantly different during Phase III and IV in southern Africa (P≥0.25).

MANOVA summary

	Df	Pillai	approx F	num Df	den Df	P
Phases	1	0.004	1.047	3	686	0.371
Residuals	700					

ANOVA summary

	Df	Sum Sq	Mean Sq	F value	P
Response Diameter:					
Phases	1	0.001	0.001	0.003	0.958
Residuals	700	296.554	0.423		
Response Thickness:					
Phases	1	0.032	0.032	0.671	0.413
Residuals	700	33.474	0.048		
Response Aperture Diameter:					
Phases	1	0.175	0.175	1.325	0.25
Residuals	700	92.681	0.132		

Supplementary Table 5. Two-way MANOVA showing significant difference in bead characteristics between eastern and southern Africa during Phase III (19-11.6 ka) (Pillai's Trace=0.63, $F_{3,62}$=34.64, P<<0.001). ANOVA performed on the MANOVA outputs shows that all parameters play a significant role in driving the regional differences in Phases III, but diameter and aperture diameter have a greater influence (P<<0.001 for both) than thickness (P=0.004).

MANOVA summary

	Df	Pillai	approx F	num Df	den Df	P	
Regression	1	0.626	34.64	3	62	2.8e-13	***
Residuals	64						

ANOVA summary

Response Diameter:

	Df	Sum Sq	Mean Sq	F value	P	
Region	1	105.29	105.289	85.406	2.147e-13	***
Residuals	64	78.90	1.233			

Response Thickness:

	Df	Sum Sq	Mean Sq	F value	P	
Region	1	0.479	0.479	9.166	0.004	*
Residuals	64	3.341	0.052			

Response Aperture Diameter:

	Df	Sum Sq	Mean Sq	F value	P	
Region	1	11.338	11.339	70.342	6.712e-12	***
Residuals	64	10.316	0.1612			

Supplementary Table 6. Two-way MANOVA showing significant difference in bead characteristics between eastern and southern Africa during Phase IV (11.6-2 ka) (Pillai's Trace=0.39, $F_{3,716}$=150.88, P<<0.001). ANOVA performed on the MANOVA outputs shows that all parameters play a significant role in driving the regional differences in Phases IV (P<<0.001 for all three parameters).

MANOVA summary

	Df	Pillai	approx F	num Df	den Df	P
Region	1	0.3873	150.88	3	716	< 2.2e-16 ***
Residuals	718					

ANOVA summary

Response Diameter:

	Df	Sum Sq	Mean Sq	F value	P
Region	1	204.39	204.39	449.6	< 2.2e-16 ***
Residuals	718	326.40	0.0444		

Response Thickness:

	Df	Sum Sq	Mean Sq	F value	P
Region	1	2.415	2.415	49.513	4.606e-12 ***
Residuals	718	35.023	0.049		

Response Aperture Diameter:

	Df	Sum Sq	Mean Sq	F value	P
Region	1	21.592	21.592	147.82	< 2.2e-16 ***
Residuals	718	104.875	0.416		

Supplementary Table 7. Two-way MANOVA showing significant difference in bead characteristics between eastern and southern African during Phase V (2 ka-present) (Pillai's Trace=0.41, $F_{3,444}$=150.88, P<<0.001). ANOVA performed on the MANOVA outputs shows that all parameters play a significant role in driving the regional differences in Phases IV (P<<0.001 for all three parameters).

MANOVA summary

	Df	Pillai	approx F	num Df	den Df	P
df1.P1$Region	1	0.41491	104.95	3	444	< 2.2e-16 ***
Residuals	446					

ANOVA summary

Response Diameter:

	Df	Sum	Sq Mean	Sq F value	P
Region	1	315.87	315.87	304.29	< 2.2e-16 ***
Residuals	446	462.97	1.038		

Response Thickness:

	Df	Sum	Sq Mean	Sq F value	P
Region	1	3.78	3.78	67.945	1.895e-15 ***
Residuals	446	24.813	0.056		

Response Aperture.Diameter:

	Df	Sum	Sq Mean	Sq F value	P
Region	1	18.983	18.983	66.643	3.363e-15 ***
Residuals	446	127.038	0.2848		

Supplementary Table 8. Two-way MANOVA showing significant difference in bead characteristics (only bead diameter and aperture diameter) between eastern and southern Africa in Phase III (19-11.6 ka) (Pillai's Trace=0.61, $F_{2,65}$=50.72, P<<0.001). ANOVA performed on the MANOVA outputs shows that both diameter and aperture diameter play a significant role in driving the regional differences in Phases IV (P<<0.001 for both).

MANOVA summary

	Df	Pillai	approx F	num Df	den Df	Pr(>F)	
Region	1	0.609	50.723	2	65	5.349e-14	***
Residuals	66						

ANOVA summary

Response Average Diameter:

	Df	Sum Sq	Mean Sq	F value	Pr(>F)	
Region	1	93.844	93.844	82.759	2.924e-13	***
Residuals	66	74.841	1.134			

Response Aperture Diameter Average:

	Df	Sum Sq	Mean Sq	F value	Pr(>F)	
Region	1	11.760	11.7596	67.656	1.046e-11	***
Residuals	66	11.472	0.1738			

Supplementary Table 9. Two-way MANOVA showing significant difference in bead characteristics (only bead diameter and aperture diameter) between eastern and southern Africa during Phase IV (11.6-2 ka) (Pillai's Trace=0.47, $F_{2,793}$=349.03, P<<0.001). ANOVA performed on the MANOVA outputs shows that both diameter and aperture diameter play a significant role in driving the regional differences in Phase IV (P<<0.001 for both).

MANOVA summary

	Df	Pillai	approx F	num Df	den Df	Pr(>F)
Region	1	0.468	349.03	2	793	< 2.2e-16***
Residuals	794					

ANOVA summary

Response Average Diameter:

	Df	Sum Sq	Mean Sq	F value	Pr(>F)
Region	1	366.07	366.07	663.45	< 2.2e-16 ***
Residuals	794	438.11	0.55		

Response Aperture Diameter Average:

	Df	Sum Sq	Mean Sq	F value	Pr(>F)
Region	1	42.012	42.012	289.77	< 2.2e-16 ***
Residuals	794	115.117	0.145		

Supplementary Table 10. Two-way MANOVA showing significant difference in bead characteristics (only bead diameter and aperture diameter) between eastern and southern Africa during Phase V (2 ka–present) (Pillai's Trace=0.41, $F_{2,448}$=155.71, P<<0.001). ANOVA performed on the MANOVA outputs shows that both diameter and aperture diameter play a significant role in driving the regional differences in Phases IV (P<<0.001 for both).

MANOVA summary

	Df	Pillai	approx F	num Df	den Df	Pr(>F)
Region	1	0.410	155.71	2	448	< 2.2e-16 ***
Residuals	449					

ANOVA summary

Response Average Diameter:

	Df	Sum Sq	Mean Sq	F value	Pr(>F)
Region	1	327.06	327.06	306.63	< 2.2e-16 ***
Residuals	449	478.92	1.07		

Response Aperture Diameter Average:

	Df	Sum Sq	Mean Sq	F value	Pr(>F)
Region	1	21.373	21.373	68.433	1.506e-15 ***
Residuals	449	140.229	0.3123		

Supplementary Table 11. Two-way MANOVA showing that there is no significant difference in bead characteristics (only bead diameter and aperture diameter) between eastern and southern Africa during Phase I (50-33 ka) (Pillai's Trace=0.15, $F_{2,36}$=3.2, P=0.052). ANOVA performed on the MANOVA outputs shows that both bead diameters and aperture diameters are not significantly different during Phase IV for the two regions (P=0.078 for diameter and P=0.015 for aperture diameter).

MANOVA summary

	Df	Pillai	approx F	num Df	den Df	P
Sub.region	1	0.15106	3.203	2	36	0.052
Residuals	37					

ANOVA summary

Response Diameter:

	Df	Sum Sq	Mean Sq	F value	P
Sub.region	1	3.350	3.350	3.284	0.078
Residuals	37	37.742	1.0201		

Response Aperture.Diameter.:

	Df	Sum Sq	Mean Sq	F value	P
Sub.region	1	1.4430	1.443	6.493	0.015
Residuals	37	8.2222	0.2222		

Supplementary Discussion 1

Bead evolution, and the uniqueness of OES beads

Beads are small, perforated embellishments that can be suspended and worn as decoration. They appear relatively recently in our history, with the use of beads being widespread only in the last 50,000 years of an archaeological record that spans more than 3.3 million. They become prolific in the archaeological record beginning with the onset of the Later Stone Age (LSA), which marks the coalescence of many complex cultural behaviours.

The oldest known beads are perforated mollusc shells from the Middle Palaeolithic / Middle Stone Age (MSA) of southern Africa, northern Africa, and the Near East (e.g.,[87,88]). These early beads are spread over a wide geographic range, but they all share a commonality: they retain the natural external shape of the shell. Some of these specimens have natural perforations, and acted as ready-made beads. The presence of smoothed edges, or wear facets within the aperture helps distinguish these from ecofacts. In these earliest examples, the species of shell dictated the outline of the ornament, with no further alteration. The shape of the shell was the shape of the bead, and even in cases where the shell has a fabricated perforation, it does not alter the silhouette of the ornament. There is no additional manufacturing involved in these examples, rather the bead is collected in its final form, with the only modification perhaps being the perforation. This is conceptually and methodically different than shaping a raw material into a bead, and an important contrast with the OES beads that emerge in the LSA.

OES beads first emerge in the tail end of the MSA[12,34], and become commonplace in the LSA. Their unique characteristics distinguish them from the previous (marine) shell beads. First, OES beads have a fully imposed shape. The resulting bead does not resemble the original ostrich egg, but rather the shell is transformed into something new. Previously the shells dictated the shape of the bead, but OES beads are extensively shaped based on the maker’s mental template. Second, and even more importantly, OES beads are the first ornaments to be mass-produced. Finally, all OES beads (from the earliest instances to those produced and sold in tourist shops today) have a degree of standardization. They have always tended to be rounded discs less than 1 cm in diameter with a central perforation. The consistency in form is present throughout OES beads in Africa and Asia[89].

What does widespread bead production imply?

The evolution of mass-produced, standardized ornaments marks a period of change in the social lives of LSA people. They are widespread and nearly ubiquitous in LSA sites, but teasing out the meaning of their evolution is tricky. Producing these durable symbols required time, resources, and effort. There are a number of potential explanations which we will outline here, including an emerging permanence of social roles, shift towards vertically stratified society, increasing population density, or maintenance of social capital.

OES beads are durable and transferable symbols, which may suggest an emerging permanence in social roles[90]. Before this, there were likely decorative elements that did not preserve, such as scarification or body painting. Using ochre to add colouring to clothing, skin and hair for symbolic purposes likely extends well into the MSA[12]. However, ochre also serves a number of functional purposes such as hafting adhesive[91,92], fire making[93], sunscreen[94], and insect repellant[95]. On the other hand, shaped beads are laborious to produce and serve no known utilitarian function. More temporary symbols (like ochre powder on the skin) or less standardized ones (like marine shells) may be better suited for coordinating small, egalitarian groups[90]. The appearance of standardized mass-produced beads could suggest that stable roles requiring long-term social differentiation were emerging in the LSA.

Consistent social roles could also be seen as the emergence of social hierarchies, necessitating items symbolic of wealth and prestige such as OES beads. At some time in the past, certain egalitarian communities shifted towards becoming vertically stratified societies with unequal distributions of wealth and power. In these groups, an individual could earn or inherit social regard, and wearing special garb (including bead embellishments) would communicate and reinforce this status with the rest of the members. One of the earliest known archaeological examples of this is from Sunghir, in Russia. This site is widely known for its elaborate burials dating from 22-24 ka, one of which contains two children, aged 9-13 years old[96]. Their burial includes lavish grave goods such as rings, bracelets and figurines carved from reindeer antler, ceremonial ivory spears, and over 13,000 hand-made beads. Estimates have suggested the beads would have taken between 3,000 and 10,000 hours to produce[97], demonstrating that these burials are a massive investment of time and resources. It would be unsustainable to invest this intensely for every group member, so this burial stands out as prestigious. The children from Sunghir are widely believed to represent the first proof of ascribed or inherited status, as earned or achieved status would have taken years or decades to develop, and the children would not have time to develop this reputation in their short lives[97]. The emergence of OES beads in the LSA of sub-Saharan Africa could indicate the rise of ascribed social status.

Increasing population density is another possible explanation for the emergence of OES beads in the LSA. This would bring people into more regular contact with unfamiliar strangers, and beads would help mediate these interactions. Using a cost-benefit perspective, researchers[98–100] have examined potential target audiences for symbolic artifact communication in four levels of social relationships. The first level includes household family and close friends who have direct, often daily communication with the wearer[100]. With this level of familiarity, the costs of producing decorative objects outweigh the benefits, and verbal information sharing would be far more efficient[98]. The next level is more socially remote, and includes distant relatives and acquaintances. These people comprise important material and emotional support systems, and do not have daily contact with the wearer[100], and at this level it is still more costly to use symbolic ornaments for communication[13,98]. The third, and most likely audience for symbolic communication then, is virtual strangers from the same culture. These people are not directly familiar with the wearer, but can decode the transmission, and would otherwise not have access to the information[13,98]. At the farthest end of the spectrum are strangers from foreign cultures. Any symbolic communication would be lost on this group, as they would not have the social constructs to decode culturally specific messages[13,98]. The third group then of culturally similar strangers most efficiently balances the costs and benefits of ornament production, and the larger this third group is, the more efficient symbolism becomes in comparison to other forms of communication[98]. With this reasoning, symbolic ornaments may have evolved to mediate increasing contact with unfamiliar people as a result of growing population size or larger social networks, highlighting the increasing frequency and importance of interactions with strangers[101].

Finally, symbols can delineate, establish, and maintain group affiliation, so bead-use could have developed in response to increasing intergroup interaction. Having access to a network of allies is an important form of social capital, which is the ability to draw benefits from membership in a social network[102]. By spending time and resources creating alliances, people invest in relationships that offer advantages such as protection, diverse marriage partners, long-distance trade opportunities, information transfer, and resource sharing in times of stress. However, maintaining extended social networks requires a high degree of communication, social maintenance, and conflict resolution. A group that cannot satisfy these demands will eventually fission, as the neglected relationships wane[103].

Symbolic items like beads can be an effective mode of communication[103,104], and thus a good way to maintain or accumulate social capital. Unlike other methods of social maintenance (such as social grooming, gestural / postural signals, or auditory cues), a symbolic item communicates effortlessly over time and space[104]. The receiver can obtain the message from some geographic or chronological distance from the sender. Further, if the information is encoded in a physical item (such as a decorative ornament),

it continuously broadcasts the message with minimal constraints on audience size. Therefore, the emergence of beads may represent an increased investment in social capital, as a way to maintain large extended social networks.

Supplementary Discussion 2

Beadwork vs single beads

In modern examples, OES beads are strung together, or affixed to a garment, for display. Ethnographic research in Africa suggests that variations in bead arrangement and patterning have cultural meanings, and these are detectable by group members[13,15,99]. Beaded garments can convey information about social position, but they also help the wearer to embody and perform their identity[107]. Therefore, the message is not contained in individual beads, but in the donning and display of a completed piece of beadwork.

Archaeologically, OES beads tend to be recovered in isolation. These individual finds have been removed from their contextual beadworks, through loss or breakage or taphonomy, and so the impact of their original display or patterning is lost to us. However, even without their larger beadwork context, these beads still contain a different type of social information, as their subtle variations in form are a natural outcome of cultural learning[17]. Individual OES beads were probably not intended to actively signal meaning, but people from the same social group will produce similar artifact styles as they practice, worth together, and learn from one another[20]. Even though individual OES beads have been removed from their original context, they still retain these cultural variations. Some previous research has provided support for this idea from modern[65,105] and archaeological[25,106] contexts, though no wide-spread modern or experimental study has been conducted.

Stylistic variation

Cultural variations, or the "differential persistence of alternative traits through time"[107], can be functional or stylistic. Variation is *functional* when alternatives confer differential success, whereas *stylistic* variations are selectively neutral so that the performance of each variant confers equal fitness[108]. In the case of OES beads, the sole purpose is visual display, so variations in aperture diameter and bead diameter have very little (if any) effect on fitness. Certainly, there is some limitation on bead sizes, as fitness would be affected if the beads were microscopically small and invisible, or so large that they could not be worn. This leaves a range of appropriate sizes within which stylistic variation can operate.

Likewise, the size of a bead's aperture is dictated by the size of the drill bit or the thickness of available string. These traits do not affect fitness, but instead reflect social customs or locally available technology, and are therefore stylistic variations. Two groups may share similarities for a variety of reasons, including convergent evolution (same adaptations from similar pressures), inheritance through ancestry (passed from elders), or horizontal transmission (through inter-group interaction)[19,109]. Cases of convergent evolution, should occur only for selectively advantageous traits, whereas neutral traits including stylistic variation will transfer through inter or intra group cultural transmission.

Selectively neutral stylistic traits are culturally encoded, and can be analyzed to help visualize social boundaries. People from the same community will tend to choose stylistic variations similar to one another, and through social learning these ideals are spread within and between neighboring groups as acceptable bead forms. The more connections shared between two communities, the more opportunities there are for exchange, and the faster their styles will homogenize[110]. By contrast, as communities diverge styles will become more dissimilar over time. Cultural drift and innovation will shape the norms of each new group[108]. People from separated populations, with no shared social connections, will generate randomized styles[108]. The more geographic or temporal distance between two groups, the more distinct their style norms should be. Today, probably very few communities are completely isolated, as it is so easy to travel long distances or to communicate with people who are far away. However, in the ancient past, connections between networks would have been constrained by distance. This is exemplified in the Isolation by Distance model[111], which has been suggested to explain Pleistocene cultural and morphological variation[1]. Therefore, regional clusters of traits may suggest isolated populations with

distinct stylistic signatures, variables shared over large distances may indicate shared relationships, and the merging of two distinct styles may represent intermittent social contact between groups.

The potential for long-distance stylistic connection

An eastern-southern African cultural network is far more expansive than previously suspected, however there is precedence for the long-distance exchange supported by ethnographic data and geochemical sourcing studies. For example, modern hunter-gatherer communities in sub-Saharan Africa use OES beads as valued trade items, and these beads may be transferred hundreds of kilometers over the span of a few years[112,113]. This transport of OES beads seems to have roots in the Late Pleistocene, as a recent study found a 33 kyr old OES bead was chemically sourced more than 300 km from where it was recovered[80]. The practice of transferring exotic materials over long-distances extends much further into the past[12], for example by 200 ka in eastern Africa where obsidian (a high-quality material) was recovered >150 km from its source[114]. Alongside the movement of physical objects, it is highly likely that social exchanges also took place such as sharing knowledge, technology, or cultural styles. Ideas could be transmitted even further than material culture, perhaps making their way from eastern to southern Africa.

Supplementary Discussion 3

Division of Phases

In this study, we analyzed OES bead characteristics from the past 50 kyr in search of patterns revealing population connections, and their association with hydroclimate shifts in Africa during the last glacial and interglacial cycles. In order to understand potential effects of climate on these patterns, we grouped the last 50-2 kyr into four periods based on major climate shifts. According to climate model simulations, eastern Africa experienced more dramatic shifts in precipitation, while southern African rainfall was more consistent throughout the entire 50 kyr (**Supplementary Figure 1**). As the goal of this study is to verify how social connectivity of two regions responded to climatic/environmental conditions in Africa, it is more appropriate to bin the phases based on eastern African climate shifts. The oldest Phase (50 -33 ka), encompasses part of Marine Isotope Stage 3 to the time of renewed ice-sheet growth. During this period, rainfall is variable but air temperatures are several degrees higher in eastern Africa[29,37,38]. Phase II (33-19 ka) begins with the time of renewed ice-sheet growth and ends at the termination of the Last Glacial Maximum (LGM). This was the driest and coolest period experienced by eastern Africa throughout the past 50 kyr, according to climate model simulations (**Supplementary Figure 1**)[37]. Phase III (19-11.6 ka) extends from the deglaciation until the onset of Holocene. During this phase, climate conditions improved in both eastern and southern Africa with rising rainfall and air temperatures. Phase IV (11.6-2 ka) includes the entire Holocene except the last 2 kyr. In this period, the climate of both southern and eastern Africa reached stable and modern levels (**Supplementary Figure 1**). The final phase, Phase V (2 ka-present), is not based on climate shift, but rather on social shift, as this marks the entry of early herders into southern Africa[21].

Supplementary Figure 1. LOVECLIM transient model output show temperature and rainfall changes in eastern Africa (22.5 -40°E, 9°N) and southern Africa (8-35°E, 20-35°S) for the last 50 kyr. The vertical gray lines denote boundaries between different phases.

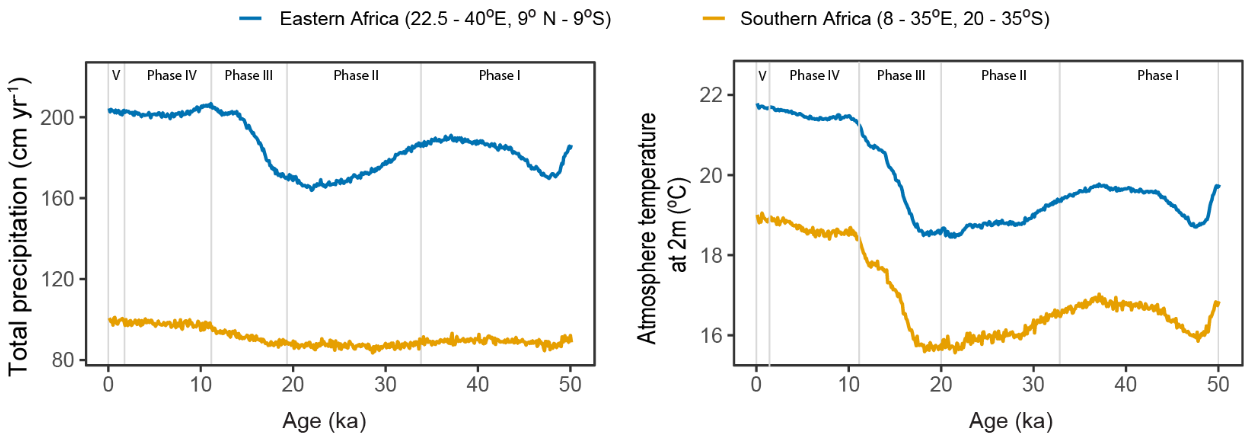

Supplementary Discussion 4

Hydroclimate changes between Phase I and II in eastern equatorial and southeast tropical Africa

The apparent cultural disconnection during Phase II between eastern and southern Africa (~33ka) coincides with hydroclimate reorganization and antiphase precipitation patterns between eastern and southeast tropical Africa. Over the past 50 kyr, rainfall and Net Primary Production (NPP) in Africa has been modulated by factors including northern hemisphere climatic changes, Indian Ocean sea surface temperatures[29], and crucially the shifting location of the Intertropical Convergence Zone (ITCZ).

Approximately ~31-29 ka, the Heinrich event III (HE3) in the North Atlantic triggered hydroclimate reorganization that affected eastern equatorial and southeast tropical Africa. During the HE3, a surge of icebergs freshened the surface waters of the northern North Atlantic, leading to a weakening of the Atlantic Meridional Overturning Circulation[115,116]. The reduced poleward heat transport caused cooling of the North Atlantic, which further spread across Eurasia[116]. This in turn weakened the interhemispheric temperature gradient, leading to a southward shift of the ITCZ.

The ITCZ movement reduced rainfall intensity in eastern Africa (eastern equatorial and the Great Lakes region), while increasing precipitation in southeast tropical Africa (the Zambezi River catchment)[29,38,45]. According to paleo rainfall records[29,35] and LOVECLIM transient climate model data[37], the African Great Lakes region entered a drying phase ~35 ka, while southeast tropical Africa experienced its wettest period from 30 to 19 ka, driven by the increased magnitude of the austral summer monsoon, and southward migration of the ITCZ[30,36,38]. In Africa, the current austral summer ITCZ position is at the northern boundary of Zambezi River (10°S). Resulting from the HE3, the ITCZ was situated between 9-20°S[30] for most of Phase II (33-19 ka). This dramatic shift is exemplified in LGM (~23-19 ka), when the ITCZ was at it southernmost point of 20°S[30,117]. This southward migration of the ITCZ, instigated by the HE3, reorganized hydroclimate patterns in eastern and southeast tropical Africa.

Zambezi River as a physical barrier between eastern and southern Africa.

The Zambezi River (~2570 km) is the longest east-flowing river in Africa. Its large catchment area (1.4 ×106 km°, ~9–20°S and ~18–37°E) geographically connects our two study regions: the Great African Lakes region, and southern Africa. As discussed above, from 30-16 ka the Zambezi River catchment experienced more intense rainfall due to the southward migration of ITCZ and stronger austral summer monsoon[30]. This enhanced rainfall could have triggered periodic flooding events, forming a geographic barrier between southern and eastern African populations.

Episodic flood events are supported by an isotopic assessment of clays deposited onto the Mozambique Shelf margin over the past 45 kyr[36]. The Mozambique Channel accumulates fine grained fluvial sediments either as discharge from the Zambezi River, or from more northerly riverine sources. By examining the sediment origin based on Neodymium (Nd) isotopic (εNd) signatures from shelf surface sediments and estimating the proportional contribution of clay content from a wide range of geographical locations in a marine sediment core, episodes with greater clay input from the Zambezi River suggests increased precipitation and periodic flooding of this large river system. Conversely, a decreased clay contribution from Zambezi river catchment indicates a decrease in rainfall or fluvial discharge.

The isotopic analysis shows consistently higher clay contribution sourced from Zambezi River from 29-16 ka, compared to that from the northern rivers[36] (**Fig. 4b**). This increased clay contribution to the Mozambique Shelf margin, broadly mirroring the increased rainfall in the Zambezi catchment at the same period[38]. This increased movement of clays and silts, resulting from the periodic flooding of the

Zambezi River, could have increased land erosion around the catchment area, thereby disturbing migration pathways and social networks between eastern and southern Africa.

Supplementary Figure 2. LOVECLIM transient model output show strong correlation between NPP and precipitation in our study regions: eastern Africa (22.5 -40°E, 9°N -9°S) and southern Africa (8-35°E, 20-35°S), suggesting that the NPP is primarily controlled by total precipitation.

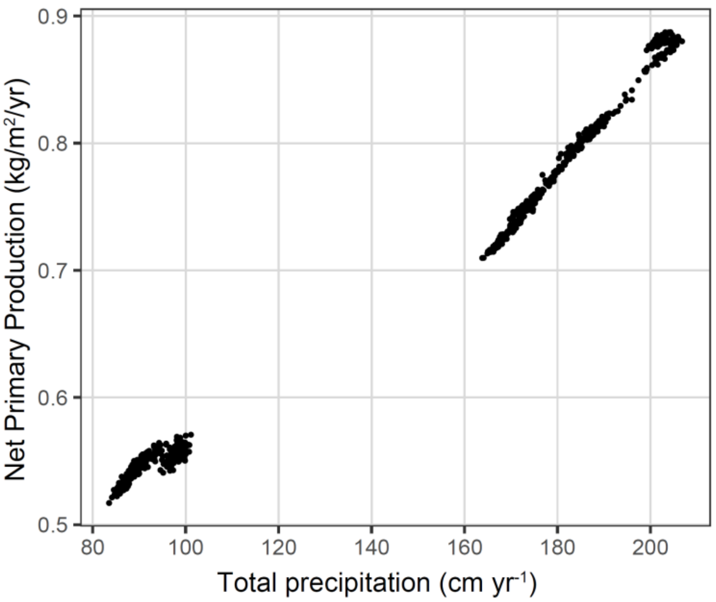

Supplementary Figure 3. LOVECLIM transient model output net primary production change in study regions: eastern Africa (22.5 -40°E, 9°N-9°S) and southern Africa (8-35°E, 20-35°S) for the last 50 kyr. The gray vertical lines denote the boundaries between different phases.

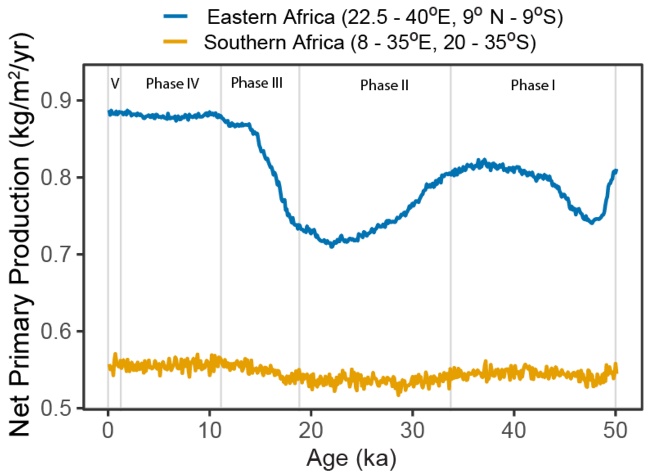

Supplementary Figure 4. LOVECLIM transient model output show Tree and grass cover in our study regions: eastern Africa (22.5 -40°E, 9°N-9°S) and southern Africa (8-35°E, 20-35°S) for the last 50 kyr. The gray vertical lines denote the boundaries between different phases.

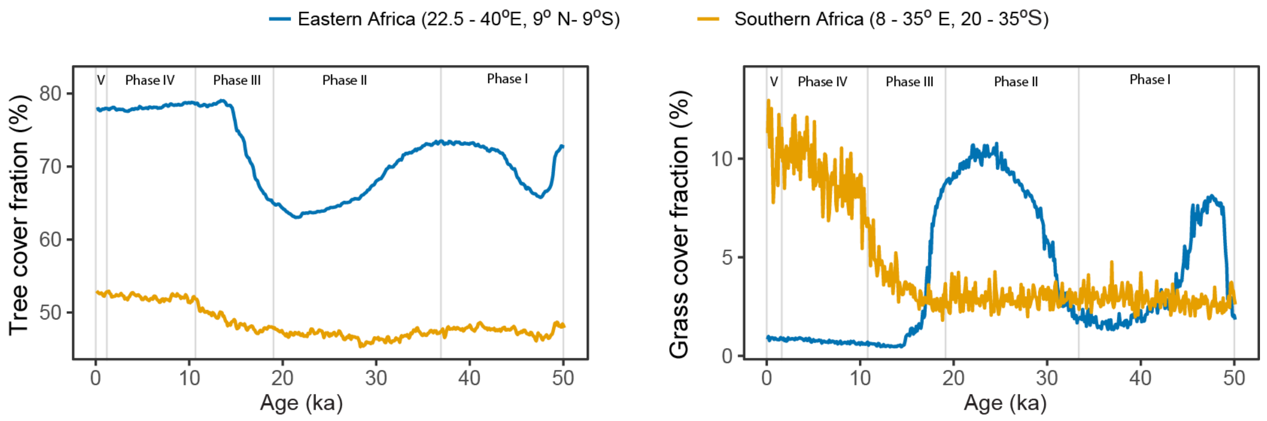

Supplementary Discussion 5

Phase II OES beads from southern Africa

The present study includes only one southern African OES bead from Phase II (33-19 ka). However, there are some commonly cited southern African sites that report small numbers of OES beads from Phases I/II (50-19 ka) including Nelson Bay Cave, Boomplaas Cave, and Kathu Pan 5. None of these collections were available for study during a data collection trip by J.M.M. in 2014. Once located, these collections should be directly dated to confirm their antiquity.

If these beads, or others of similar age in southern Africa are examined, and found to be intermediately sized (between the larger size in Phase I and the smaller Phase III), then they hint at an *in situ* development of the southern African small diameter beads. Such a shift could be due to any number of factors including changing preferences, transmission errors, limited availability of raw material, or the influence of new people entering the area. However, if Phase II (33-19 ka) OES beads are confirmed to be absent, or only appear exclusively in the smaller bead style, then this supports an independent reinvention or revival of OES bead technology in Phase III (19-11.6 ka), which may correspond to the increased rainfall and net primary precipitation that begins ~19 ka. The general lack of Phase II southern African OES beads could mark the loss of a cultural practice that was more costly than beneficial. At Border Cave, there are no OES beads in overlying layers, hinting that this tradition was not a regular part of the cultural repertoire. However, one Border Cave bead has a smaller diameter (4.3 mm), which could suggest ancient roots for the diminutive bead tradition. This patchy development of cultural practices seems to be evident in other archaeological evidence from this time, which shows a staggered technological transition with possible co-existing but culturally unique sub-populations in southern Africa[46,47]. Future OES bead data from Phase II in southern Africa is warranted to test hypotheses about the mechanism of change or potential absence of bead use.

Boomplaas Cave

Boomplaas Cave is located in South Africa, and gained special attention for its collection of painted stones found in association with Stone Age occupations. A team studying environmental and cultural change in the Late Quaternary of the southern Cape first investigated the site. This project, based at the University of Stellenbosch, excavated a 1 m^2 test unit in 1974, and found five metres of well stratified deposition spanning the last 80,000 years[118].

The most pertinent OES beads from the Boomplaas Cave collection come from two particular levels. First, the CL (carbonized loam) layer, found approximately 210-240 cm below surface, is important. This layer has a complex stratigraphy with anthropogenic ash lenses, and appears to be a phase of intense site use[119]. The base of CL was radiocarbon dated to 14,200 ± 240 BP[118]. Artifacts from this layer include micro-bladelets, small pyramidal cores, bone points, decorated water container fragments, and beads, all assigned to the Robberg Industry[120]. The beads from the CL layer are reported as being made from both bone and OES. There are marine shell beads present in the overlying layers, however none were recovered from CL. The second important layer is the OLP layer, which is found approximately 340-390 cm below surface and has two OES artifacts reported[120]. When subjected to dating, this layer was beyond the upper radiocarbon limit, suggesting a minimum age of 40,000 years. If the two artifacts from OLP are stratigraphically intact, then they are among the earliest known OES beads.

Ultimately, J.M.M. was unable to study the Boomplaas collection. The material is stored at the Iziko Museum in Cape Town, South Africa, and after receiving permission to study the collection, J.M.M. travelled to Cape Town in the summer of 2014 for this purpose. Upon arrival, J.M.M. was provided with several boxes labelled as containing OES from Boomplaas, however there were no beads or preforms in any of the boxes, only unmodified OES. The curators were unable to locate any Boomplaas beads. The

museum had been undergoing renovations, and J.M.M. was given lab space in the Slave Museum rather than the main building. This may have contributed to the difficulties in locating the assemblage. We suspect that the delicate beads were stored with other small finds rather than the kilograms of unmodified OES, so the box could not easily be identified from the label. The Boomplaas OES bead collection remains unstudied, but should be strongly considered in the future, including direct dating of the OLP artifacts to rule out stratigraphic mixing.

Kathu Pan 5

Kathu Pan 5 (KP5) is a site in South Africa which reportedly has OES beads from early LSA layers dated between 19,800 ± 280 and 32,100 ± 780 BP[84]. The collection is stored at the McGregor Museum in Kimberley, South Africa. Unfortunately, the curator and J.M.M. were unable to locate any OES beads from the site. J.M.M. personally scoured the storage room, and examined every single box from the site twice over, but none had any beads. The boxes contained lithics, bone, sediment samples, and some small amounts of unmodified OES. Some of the bags were empty, with tags inside saying the contents were "sent to Vogel" in 1988 for analysis. It is possible that these items were OES beads, as the tags probably refer to Johann Carl Vogel who was a well-known dating specialist in South Africa. However, it seems it is unlikely that these missing objects were beads, given the small amount of unmodified OES combined with the fact that no other bags contained beads, and there were no references to beads on artifact tags.

A staff member at the MacGregor Museum suggested that the publication of beads from KP5 might be erroneous. The finds are reported in a larger summary of test excavations from sites in the Kathu region, written by Dr. Peter Beaumont. His co-authored book (written with Dr. David Morris) summarizes excavation work that he oversaw between 1979 and 1990, from eleven archaeological sites. Descriptions of the cultural sequence list anecdotally that OES beads are found at Kathu Pan 1 and 5, although no table of finds is included, and no unpublished reports were cited. Beaumont writes that two OES beads from Kathu Pan 1, Stratum 3, were submitted for direct radiocarbon dating, but I can find no mention of reported dates. All later references to early OES beads from Kathu Pan (e.g.,[121,122]) reference back to the Beaumont and Morris[84] literature.

Nelson Bay Cave

Nelson Bay Cave (NBC) is a large cavern measuring approximately 30 x 15 m, located 550 km to the east of Cape Town, South Africa. The cave deposits were looted in the late 1800s and early 1900s to provide artifacts for museums[123], and the first archaeological work took place between 1964 and 1979[76]. No further excavation has been conducted at the site since that time. However, some restoration work has taken place to secure the open excavation units against erosion[123].

Study at NBC has largely focused on the Holocene occupation levels, but there is some mention of Pleistocene-aged OES beads. Deacon's publication has a figure of a line drawing of four beads, with the caption "ostrich eggshell beads from Nelson Bay dated *c*. 18500 BP[124]. In addition, there is mention in the faunal analysis that ostrich eggshell fragments were recovered from the Yellow-Grey Gritty Loam layer (dated to 18 ka) on upwards[125], although it is not clear if there are beads present in this lowest layer. As I was already heading to the Iziko Museum in Cape Town to study the Boomplaas assemblage, I decided it was worthwhile to also examine the NBC collection.

Unfortunately, no OES artifacts from Pleistocene layers could be located during research at the Iziko museum in 2014. There were fourteen preforms and 535 OES beads from Holocene levels, all of which were analyzed instead.

Supplementary Discussion 6

Recommendations for collecting and reporting future OES bead data

Our study highlights that even a cursory analysis of OES beads can contribute important information to understand sub-population connections and cultural exchange. However, in order to continue investigating OES bead variation, consistent and standardized collection of data is required. We suggest researchers move beyond a traditional reporting presence/absence of OES beads with some limited analysis. In this study, we used three metric variables (bead diameter, aperture diameter, thickness) to reveal regional patterns, though refer to Miller[69] for a more comprehensive list of qualitative variables. Since PCA revealed that bead diameter is the most significant of these three metrics, we suggest that at minimum bead diameters should be measured and reported. Further, individual bead characteristics should be included in publications, rather than averaged data by excavation level. As demonstrated in Miller and Sawchuk[21], providing only average diameters served to masked nuances that documented social responses to the introduction of herding. Measurements of individual beads are particularly important for Pleistocene assemblages where there is already very little data available.

We also recommend the responsible direct dating of selected OES artifacts, to confirm their antiquity. OES beads are small, often less than 1 cm in diameter, with colouring similar to sediment, both of which make them tricky to recover *in situ*. Further, OES beads are lightweight and can easily be dislodged during the excavation process. It can therefore be difficult to tell whether isolated finds are intrusive, or genuinely ancient examples. We therefore suggest, particularly for Pleistocene-aged assemblages, that OES beads be considered for radiocarbon dating. It will become increasingly important to refine the chronology as more data is added. Destructive analysis should only be considered after thorough documentation is complete.

Existing debates about bead size (as discussed in this paper) are relevant only for beads that have reached the end stage of manufacture, so it is important to distinguish and record the production value of each specimen. There are two commonly used systems for recording production value (Kandel and Conard's[63] and Orton's[48], each provides different benefits. Kandel and Conard's scheme works well with statistics software I use, as the categories are all numerical. Orton's system is important because it records the presence of different manufacture Pathways. There may be a few instances in which the two schemes did not agree on whether the specimen was a completed bead, and in these cases, we suggest using Orton's system as the deciding factor. Orton distinguishes completed beads from those still in the production sequence by the presence of "use polish"[48], while Kandel and Conard[63] suggest that the form of the bead (specifically how finely shaped it is) indicates the end stage. Orton does not further define what use polish looks like, or where it should occur, so we have taken it to mean smoothing / patina around the outer rim and / or within the aperture at the position of restriction. In this paper, any bead with such smoothing or patina present is deemed as completed, even if it was not finely shaped.

As a best practice for creating a record of OES beads and preforms, a minimum of three photos of each specimen should be captured (see examples in **Supplementary Figure 5**, below). This can be achieved with a digital microscope, an adjustable height stand, and a digital scale. The three pictures should be taken vertically (top down) rather than at an oblique angle, and consist of the cuticle surface, the mammillary surface, and at least one picture of the profile view. The profile photo can be taken by holding the specimen gently with nylon tipped tweezers, one tong against the cuticle surface and the other against the mammillary surface, and turning the bead so the edge of the shell is towards the camera. For consistency, the profile photo should always be taken in the same orientation, with the cuticle surface of the shell towards the top of the image. The cuticle and mammillary surface photos should have a photo scale visible in the picture, as this scale will allow accurate digital measurements to be taken later of the aperture diameter. Using the photo scale as a guide, measurements can be taken in the freeware program ImageJ. Note this technique does not allow thickness measurements to be recreated from the pictures.

Supplementary Figure 5: Example of OES bead inventory photos; cuticle surface on left, mammillary surface in center, shell profile on right.

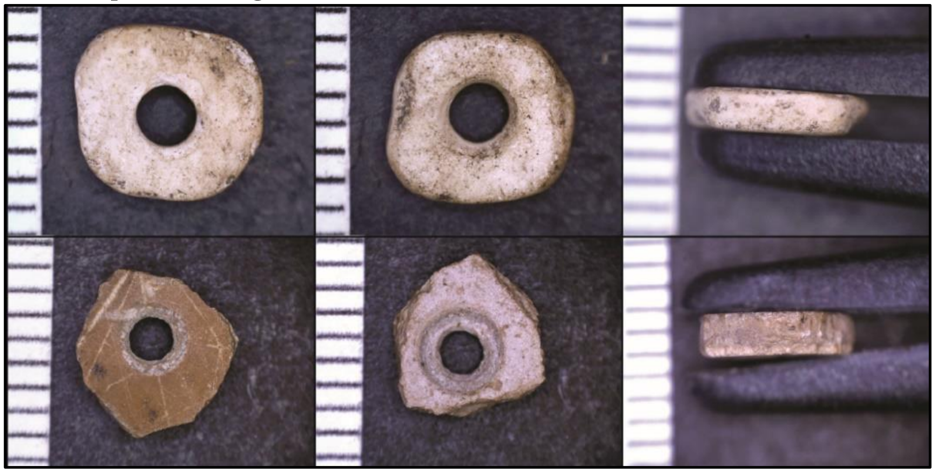

Measurements of bead/preform diameter and shell thickness can be recorded with calipers. We typically record the highest and lowest diameter measurements, and calculate an average value. When a completed but broken bead had at least 50% of its circumference remaining, we record the maximum width, and use that instead of average diameter. Some researchers have commented that plastic calipers are better to use than metal calipers, as they are less likely to break breads during analysis. We exclusively used metal tipped calipers and did not experience breakage while measuring thickness or diameter. However, if plastic calipers are available, then it seems wise to use them. Under no circumstances should calipers be used to measure aperture diameter.

Supplementary References

1. Scerri, E. M. L. *et al.* Did our species evolve in subdivided populations across Africa, and why does it matter? *Trends Ecol. Evol.* **33**, 582–594 (2018).

12. McBrearty, S. & Brooks, A. S. The revolution that wasn't: a new interpretation of the origin of modern human behavior. *J. Hum. Evol.* **39**, 453–563 (2000).

13. Kuhn, S. L. & Stiner, M. C. Body ornamentation as information technology: towards and understanding of the significance of early beads. in *Rethinking the Human Revolution: New Behavioural and Biological Perspectives on the Origin and Dispersal of Modern Humans* (eds. Mellars, P., Boyle, K., Bar-Yosef, O. & Stringer, C.) 45–54 (Oxbow Books, 2007).

14. d'Errico, F. *et al.* Early evidence of San material culture represented by organic artifacts from Border Cave, South Africa. *Proc. Natl. Acad. Sci.* **109**, 13214–13219 (2012).

15. d'Errico, F. & Vanhaeren, M. Upper Palaeolithic mortuary practices: reflection of ethnic affiliation, social complexity, and cultural turnover. in *Death Rituals, Social Order and the Archaeology of Immortality in the Ancient World* (eds. Renfrew, C., Boyd, M. J. & Morley, I.) 45–62 (Cambridge University Press, 2016).

17. Sackett, J. R. Approaches to style in lithic archaeology. *J. Anthropol. Archaeol.* **1**, 59–112 (1982).

19. Crema, E. R., Kerig, T. & Shennan, S. Culture, space, and metapopulation: a simulation-based study for evaluating signals of blending and branching. *J. Archaeol. Sci.* **43**, 289–298 (2014).

20. Wenger, E. *Communities of Practice: Learning, Meaning, and Identity*. (Cambridge University Press, 1998).

21. Miller, J. M. & Sawchuk, E. A. Ostrich eggshell bead diameter in the Holocene: regional variation with the spread of herding in eastern and southern Africa. *PLoS ONE* **14**, e0225143 (2019).

22. Jacobson, L. The size variability of ostrich eggshell beads from central Namibia and its relevance as a stylistic and temporal marker. *S. Afr. Archaeol. Bull.* **42**, 55–58 (1987).

24. Dayet, L., Erasmus, R., Val, A., Feyfant, L. & Porraz, G. Beads, pigments and early Holocene ornamental traditions at Bushman Rock Shelter, South Africa. *J. Arch. Sci. Rep.* **13**, 635–651 (2017).

25. d'Errico, F. *et al.* Trajectories of cultural innovation from the Middle to Later Stone Age in eastern Africa: personal ornaments, bone artifacts, and ocher from Panga Ya Saidi, Kenya. *J. Hum. Evol.* **141**, 102737 (2020).

26. Tryon, C. A. *et al.* Middle and Later Stone Age chronology of Kisese II Rockshelter (UNESCO World Heritage Kondoa Rock-Art Sites), Tanzania. *PLoS ONE* **13**, e0192029 (2018).

27. Collins, B., Wojcieszak, M., Nowell, A., Hodgskiss, T. & Ames, C. J. H. Beads and bead residues as windows to past behaviours and taphonomy: a case study from Grassridge Rockshelter, eastern Cape, South Africa. *Archaeol. Anthropol. Sci.* **12**, 192 (2020).

29. Tierney, J. E. *et al.* Northern hemisphere controls on tropical southeast African climate during the past 60,000 years. *Science* **322**, 252–255 (2008).

30. Wang, Y. V. *et al.* Northern and southern hemisphere controls on seasonal sea surface temperatures in the Indian Ocean during the last deglaciation. *Paleoceanography* **28**, 619–632 (2013).

34. Miller, J. M. & Willoughby, P. R. Radiometrically dated ostrich eggshell beads from the Middle and Later Stone Age of Magubike Rockshelter, southern Tanzania. *J. Hum. Evol.* **74**, 118–122 (2014).

36. van der Lubbe, H. J. L., Frank, M., Tjallingii, R. & Schneider, R. R. Neodymium isotope constraints on provenance, dispersal, and climate-driven supply of Zambezi sediments along the Mozambique Margin during the past ∼45,000 years. *Geochem. Geophys. Geosyst.* **17**, 181–198 (2016).

37. Timmermann, A. & Friedrich, T. Late Pleistocene climate drivers of early human migration. *Nature* **538**, 92–95 (2016).

38. Wang, Y. V. *et al.* What does leaf wax δD from a mixed C3/C4 vegetation region tell us? *Geochimica et Cosmochimica Acta* **111**, 128–139 (2013).

45. Ziemen, F. A., Kapsch, M.-L., Klockmann, M. & Mikolajewicz, U. Heinrich events show two-stage climate response in transient glacial simulations. *Clim. Past* **15**, 153–168 (2019).

46. Mackay, A., Stewart, B. A. & Chase, B. M. Coalescence and fragmentation in the late Pleistocene archaeology of southernmost Africa. *J. Hum. Evol.* **72**, 26–51 (2014).

47. Bousman, C. B. & Brink, J. S. The emergence, spread, and termination of the Early Later Stone Age event in South Africa and southern Namibia. *Quat. Int.* **495**, 116–135 (2018).

48. Orton, J. Later Stone Age ostrich eggshell bead manufacture in the Northern Cape, South Africa. *J. Archaeol. Sci.* **35**, 1765–1775 (2008).

54. Wendt, W. E. 'Art mobilier' from the Apollo 11 Cave, South West Africa: Africa's oldest dated works of art. *S. Afr. Archaeol. Bull.* **31**, 5–11 (1976).

55. Vogelsang, R. *et al.* New excavations of Middle Stone Age deposits at Apollo 11 Rockshelter, Namibia: Stratigraphy, archaeology, chronology and past environments. *J. Afr. Archaeol.* **8**, 185–218 (2010).

56. Murray-Wallace, C. V., Richter, J. & Vogelsang, R. Aminostratigraphy and taphonomy of ostrich eggshell in the sedimentary infill of Apollo 11 Rockshelter, Namibia. *J. Archaeol. Sci. Rep.* **4**, 143–151 (2015).

57. Villa, P. *et al.* Border Cave and the beginning of the Later Stone Age in South Africa. *Proc. Natl. Acad. Sci.* **109**, 13208–13213 (2012).

58. Prendergast, M. E. *et al.* Pastoral Neolithic sites on the southern Mbulu Plateau, Tanzania. *Azania* **48**, 498–520 (2013).

59. Humphreys, A. J. B. A preliminary report on test excavations at Dikbosch Shelter I, Herbert District, Northern Cape. *S. Afr. Archaeol. Bull.* **29**, 115 (1974).

60. Ambrose, S. H. Chronology of the Later Stone Age and food production in East Africa. *J. Archaeol. Sci.* **25**, 377–392 (1998).

61. Jacobson, L. More on ostrich eggshell bead size variability: The geduld early herder assemblage. *S. Afr. Archaeol. Bull.* 174–175 (1987).

62. Smith, A. B., Yates, R., Miller, D., Jacobson, L. & Evans, G. Excavations at Geduld and the appearance of early domestic stock in Namibia. *S. Afr. Archaeol. Bull.* **50**, 3–20 (1995).

63. Kandel, A. W. & Conard, N. J. Production sequences of ostrich eggshell beads and settlement dynamics in the Geelbek Dunes of the Western Cape, South Africa. *J. Archaeol. Sci.* **32**, 1711–1721 (2005).

64. Collins, B., Wilkins, J. & Ames, C. Revisiting the Holocene occupations at Grassridge Rockshelter, eastern Cape, South Africa. *S. Afr. Archaeol. Bull.* **72**, 162–170 (2017).

65. Smith, A. B., Sadr, K., Gribble, J. & Yates, R. Excavations in the South-Western Cape, South Africa, and the archaeological identity of prehistoric hunter-gatherers within the last 2000 years. *S. Afr. Archaeol. Bull.* **46**, 71–91 (1991).

66. Sadr, K., Smith, A., Plug, I., Orton, J. & Mutti, B. Herders and foragers on Kasteelberg: Interim report of excavations 1999-2002. *S. Afr. Archaeol. Bull.* **58**, 27–32 (2003).

67. Pleurdeau, D. *et al.* "Of sheep and men": Earliest direct evidence of caprine domestication in southern Africa at Leopard Cave (Erongo, Namibia). *PLoS ONE* **7**, e40340 (2012).

68. Werner, J. J. & Willoughby, P. R. Middle Stone Age technology and cultural evolution at Magubike Rockshelter, southern Tanzania. *Afr. Archaeol. Rev.* **34**, 249–273 (2017).

69. Miller, J. M. Variability in ostrich eggshell beads from the Middle and Later Stone Age of Africa. (University of Alberta, 2019).

70. Miller, J. M. The ostrich eggshell beads of Mlambalasi Rockshelter, southern Tanzania. (University of Alberta, 2012).

71. Sawchuk, E. A. & Willoughby, P. R. Terminal Pleistocene Later Stone Age human remains from the Mlambalasi Rock Shelter, Iringa Region, southern Tanzania. *Int. J. Osteoarchaeol.* **25**, 593–607 (2015).

72. Biittner, K. M. *et al.* Excavations at Mlambalasi Rockshelter: A terminal Pleistocene to recent Iron Age record in southern Tanzania. *Afr. Archaeol. Rev.* **34**, 275–295 (2017).

73. Mehlman, M. J. Later Quaternary archaeological sequences in northern Tanzania. (University of Illinois, 1989).

74. Prendergast, M. E. *et al.* New excavations at Mumba Rockshelter, Tanzania. *J. Afr. Archaeol.* **5**, 217–44 (2007).

75. Gliganic, L. A., Jacobs, Z., Roberts, R. G., Domínguez-Rodrigo, M. & Mabulla, A. Z. P. New ages for Middle and Later Stone Age deposits at Mumba Rockshelter, Tanzania: Optically Stimulated Luminescence dating of quartz and feldspar grains. *J. Hum. Evol.* **62**, 533–547 (2012).

76. Inskeep, R. R. & Vogel, J. C. Radiocarbon dates from the Holocene levels at Nelson Bay Cave, and an interim report on their associations. *S. Afr. Archaeol. Bull.* **40**, 103 (1985).

77. Inskeep, R. R. *Nelson Bay Cave, Cape Province, South Africa: The Holocene levels*. (British Archaeological Reports, 1987).

78. Shipton, C. *et al.* 78,000-year-old record of Middle and Later Stone Age innovation in an East African tropical forest. *Nat. Commun.* **9**, 1832 (2018).

79. Orton, J., Hart, T. & Halkett, D. Shell middens in Namaqualand: Two Later Stone Age sites at Rooiwalbaai, Northern Cape Province, South Africa. *S. Afr. Archaeol. Bull.* 24–32 (2005).

80. Stewart, B. A. *et al.* Ostrich eggshell bead strontium isotopes reveal persistent macroscale social networking across late Quaternary southern Africa. *Proc. Natl. Acad. Sci.* **117**, 6453–6462 (2020).

81. Steele, T. E., Mackay, A. & Fitzsimmons, K. E. Varsche Rivier 003: a Middle and Later Stone Age Site with Still Bay and Howiesons Poort assemblages in southern Namaqualand, South Africa. *PaleoAnthropol.* **102**, 100–163 (2016).

82. Robbins, L. H. Direct dating of worked ostrich eggshell in the Kalahari. *Nyame Akuma* **52**, 11–16 (1999).

83. Robbins, L. H. *et al.* Archaeology, palaeoenvironment, and chronology of the Tsodilo Hills White Paintings Rock Shelter, northwest Kalahari Desert, Botswana. *J. Archaeol. Sci.* **27**, 1085–1113 (2000).

84. Beaumont, P. B. & Morris, D. *Guide to archaeological sites in the Northern Cape*. (McGregor Museum, 1990).

85. Thackeray, A. I., Thackeray, J. F., Beaumont, P. B. & Vogel, J. C. Dated rock engravings from Wonderwerk Cave, South Africa. *Science, New Series* **214**, 64–67 (1981).

86. Lee-Thorp, J. A. & Ecker, M. Holocene environmental change at Wonderwerk Cave, South Africa: Insights from stable light isotopes in ostrich eggshell. *Afr. Archaeol. Rev.* **32**, 793–811 (2015).

87. Bouzouggar, A. *et al.* 82,000-year-old shell beads from North Africa and implications for the origins of modern human behavior. *Proc. Natl. Acad. Sci.* **104**, 9964–9969 (2007).

88. d'Errico, F., Henshilwood, C., Vanhaeren, M. & van Niekerk, K. Nassarius kraussianus shell beads from Blombos Cave: Evidence for symbolic behaviour in the Middle Stone Age. *J. Hum. Evol.* **48**, 3–24 (2005).

89. Wang, C., Zhang, Y., Gao, X., Zhang, X. & Wang, H. Archaeological study of ostrich eggshell beads collected from SDG site. *Chinese Sci. Bull.* **54**, 3887–3895 (2009).

90. Kuhn, S. L. Signaling Theory and technologies of communication in the Paleolithic. *Biol. Theory* **9**, 42–50 (2014).

91. Wadley, L., Hodgskiss, T. & Grant, M. Implications for complex cognition from the hafting of tools with compound adhesives in the Middle Stone Age, South Africa. *Proc. Natl. Acad. Sci.* **106**, 9590–9594 (2009).

92. Zipkin, A. M., Wagner, M., McGrath, K., Brooks, A. S. & Lucas, P. W. An experimental study of hafting adhesives and the implications for compound tool technology. *PLoS ONE* **9**, e112560 (2014).

93. Heyes, P. J. *et al.* Selection and use of manganese dioxide by Neanderthals. *Sci. Rep.* **6**, 22159 (2016).

94. Rifkin, R., d'Errico, F., Dayet-Boulliot, L. & Summers, B. Assessing the photoprotective effects of red ochre on human skin by in vitro laboratory experiments. *S. Afr. J. Sci.* **111**, 1–8 (2015).

95. Rifkin, R. Ethnographic and experimental perspectives on the efficacy of ochre as a mosquito repellent. *S. Afr. Archaeol. Bull.* **70**, 64–75 (2015).

96. Pettitt, P. B. & Bader, N. O. Direct AMS Radiocarbon dates for the Sungir mid Upper Palaeolithic burials. *Antiquity* **74**, 269–270 (2000).

97. Trinkaus, E., Buzhilova, A. P., Mednikova, M. B. & Dobrovolskava, M. V. *The People of Sunghir: Burials, Bodies, and Behavior in the Earlier Upper Paleolithic*. (Oxford University Press, 2014).

98. Wobst, H. M. Stylistic Behavior and Information Exchange. in *For the Director: Research Essays in Honor of James B. Griffin* (eds. Wobst, H. & Cleland, C. E.) (University of Michigan, 1977).

99. Kuhn, S. L. & Stiner, M. C. Paleolithic ornaments: Implications for cognition, demography and identity. *Diogenes* **54**, 40–48 (2007).

100. Gamble, C. Palaeolithic society and the release from proximity: A network approach to intimate relations. *World Archaeol.* **29**, 426–449 (1998).

101. Rossano, M. J. Making friends, making tools, and making symbols. *Curr. Anthropol.* **51**, S89–S98 (2010).

102. Portes, A. Social capital: its origins and applications in modern sociology. *Ann. Rev. Sociol.* **24**, 1–24 (1998).

103. Dunbar, R. I. M. Coevolution of neocortical size, group size and language in humans. *Behav. Brain Sci.* **16**, 681–694 (1993).

104. Coward, F. & Gamble, C. Big brains, small worlds: material culture and the evolution of the mind. *Phil. Trans. R. Soc. B* **363**, 1969–1979 (2008).

105. Wilmsen, E. N. Ostrich eggshells and their beads. *S. Afr. Archaeol. Bull.* **70**, 89–105 (2015).

106. Wei, Y. *et al.* A technological and morphological study of Late Paleolithic ostrich eggshell beads from Shuidonggou, North China. *J. Archaeol. Sci.* **85**, 83–104 (2017).

107. Teltser, P. A. Culture history, evolutionary theory, and frequency seriation. in *Evolutionary Archaeology: Methodological Issues* (eds. Skibo, J. & Teltser, P. A.) 51–68 (University of Arizona Press, 1995).

108. Neiman, F. D. Stylistic variation in evolutionary perspective: inferences from decorative diversity and interassemblage distance in Illinois Woodland ceramic assemblages. *Am. Antiq.* **60**, 7–36 (1995).

109. Collard, M., Shennan, S. J. & Tehrani, J. J. Branching, blending, and the evolution of cultural similarities and differences among human populations. *Evol. Hum. Behav.* **27**, 169–184 (2006).

110. Borgatti, S. P., Mehra, A., Brass, D. J. & Labianca, G. Network analysis in the social sciences. *Science* **323**, 892–895 (2009).

111. Wright, S. Isolation by distance. *Genetics* **28**, 114–138 (1943).

112. Lee, R. B. *The Dobe! Kung: Case Studies in Cultural Anthropology*. (Holt, Rinehart and Winston, 1984).

113. Mitchell, P. Anyone for Hxaro? Thoughts on the theory and practice of exchange in southern African Later Stone Age archaeology. in *Researching Africa's Past: New Contributions from British Archaeologists* (eds. Haour, A. & Hobart, J. H.) vol. 57 35–43 (Oxford School of Archaeology, 2003).

114. Blegen, N. The earliest long-distance obsidian transport: evidence from the ~200 ka Middle Stone Age Sibilo School Road Site, Baringo, Kenya. *J. Hum. Evol.* **103**, 1–19 (2017).

115. Henry, L. G. *et al.* North Atlantic ocean circulation and abrupt climate change during the last glaciation. *Science* **353**, 470–474 (2016).

116. Rahmstorf, S. Ocean circulation and climate during the past 120,000 years. *Nature* **419**, 207–214 (2002).

117. Verschuren, D. *et al.* Half-precessional dynamics of monsoon rainfall near the East African Equator. *Nature* **462**, 637–641 (2009).

118. Deacon, H. J., Deacon, J. & Brooker, M. Four painted stones from Boomplaas Cave, Oudtshoorn District. *S. Afr. Archaeol. Bull.* **31**, 141 (1976).

119. Deacon, H. J. Excavations at Boomplaas Cave - A sequence through the Upper Pleistocene and Holocene in South Africa. *World Archaeol.* **10**, 241–257 (1979).

120. Deacon, H. J. Two Late Pleistocene-Holocene archaeological depositories from the Southern Cape, South Africa. *S. Afr. Archaeol. Bull.* **50**, 121 (1995).

121. Wadley, L. The Pleistocene Later Stone Age South of the Limpopo River. *J World Prehist* **7**, 243–296 (1993).

122. Beaumont, P. B. & Bednarik, R. G. Tracing the emergence of palaeoart in sub-Saharan Africa. *Rock Art Res.* **30**, 33 (2013).

123. Deacon, J. & Brett, M. Peeling away the past: The display of excavations at Nelson Bay Cave. *S. Afr. Archaeol. Bull.* **48**, 98 (1993).

124. Deacon, J. Changes in the archaeological record in South Africa at 18,000 BP. in *The World at 18,000: Low Latitudes* (eds. Soffer, O. & Gamble, C.) vol. 2 170–188 (Unwin Hyman, 1990).

125. Klein, R. G. Preliminary report on the July through September 1970 excavations at Nelson Bay Cave, Plettenberg Bay (Cape Province, South Africa). in *Palaeoecology of Africa* (eds. Van Zinderen Bakker, E. M. & Coetzee, J. A.) vol. 6 177–208 (A. A. Balkema, 1972).
