# Supplementary material for: Ostrich eggshell beads reveal 50,000-year-old social network in Africa
Source: Nature. 2021 Dec 20;601(7892):234–9. doi: 10.1038/s41586-021-04227-2 (PMC8755535; doi:10.1038/s41586-021-04227-2)
Supplement: Supplementary file 2 — Reporting Summary [file 41586_2021_4227_MOESM2_ESM.pdf]

Corresponding author(s): Miller, Jennifer; Wang, Yiming

Last updated by author(s): Oct 26, 2021

Reporting Summary

Nature Portfolio wishes to improve the reproducibility of the work that we publish. This form provides structure for consistency and transparency in reporting. For further information on Nature Portfolio policies, see our Editorial Policies and the Editorial Policy Checklist.

Statistics

For all statistical analyses, confirm that the following items are present in the figure legend, table legend, main text, or Methods section.

n/a	Confirmed	
☐	☒	The exact sample size (*n*) for each experimental group/condition, given as a discrete number and unit of measurement
☐	☒	A statement on whether measurements were taken from distinct samples or whether the same sample was measured repeatedly
☐	☒	The statistical test(s) used AND whether they are one- or two-sided<br>*Only common tests should be described solely by name; describe more complex techniques in the Methods section.*
☐	☒	A description of all covariates tested
☐	☒	A description of any assumptions or corrections, such as tests of normality and adjustment for multiple comparisons
☐	☒	A full description of the statistical parameters including central tendency (e.g. means) or other basic estimates (e.g. regression coefficient) AND variation (e.g. standard deviation) or associated estimates of uncertainty (e.g. confidence intervals)
☐	☒	For null hypothesis testing, the test statistic (e.g. F, t, r) with confidence intervals, effect sizes, degrees of freedom and P value noted<br>*Give P values as exact values whenever suitable.*
☒	☐	For Bayesian analysis, information on the choice of priors and Markov chain Monte Carlo settings
☒	☐	For hierarchical and complex designs, identification of the appropriate level for tests and full reporting of outcomes
☒	☐	Estimates of effect sizes (e.g. Cohen's *d*, Pearson's *r*), indicating how they were calculated

Our web collection on statistics for biologists contains articles on many of the points above.

Software and code

Policy information about availability of computer code

Data collection	n/a
Data analysis	All statistical analyses were performed in R version 4.0.151 with RStudio interface version 1.3.959.

For manuscripts utilizing custom algorithms or software that are central to the research but not yet described in published literature, software must be made available to editors and reviewers. We strongly encourage code deposition in a community repository (e.g. GitHub). See the Nature Portfolio guidelines for submitting code & software for further information.

Data

Policy information about availability of data

All manuscripts must include a data availability statement. This statement should provide the following information, where applicable:

- Accession codes, unique identifiers, or web links for publicly available datasets
- A description of any restrictions on data availability
- For clinical datasets or third party data, please ensure that the statement adheres to our policy

All data generated or analyzed for this study are included in this published article (and Supplementary Table 1). The data and R code are available from GitHub.

Field-specific reporting

Please select the one below that is the best fit for your research. If you are not sure, read the appropriate sections before making your selection.

☐ Life sciences ☒ Behavioural & social sciences ☐ Ecological, evolutionary & environmental sciences

For a reference copy of the document with all sections, see nature.com/documents/nr-reporting-summary-flat.pdf

Behavioural & social sciences study design

All studies must disclose on these points even when the disclosure is negative.

Study description	This is a quantitative study of archaeological artifacts (ostrich eggshell beads) from various sites in sub-Saharan Africa. Analysis was non-destructive, and no new excavation was conducted for this study.
Research sample	This study aims to assess bead morphological variation from the Late Pleistocene to Holocene, therefore we targeted assemblages with the oldest securely dated beads, well-dated sequences in each region (eastern and southern Africa), and sites with available data. Each assemblage is representative of the site from which it derives, and sites are representative of each region.
Sampling strategy	We compiled data from 31 sites in eastern (22.5-40°E, 9°N-9°S) and southern Africa (8-35°E, 20-35°S), totaling 1516 individual beads. All completed beads from each site were analysed, so the sample size is all beads recovered during excavation. This is the maximum possible data, and is therefore an appropriate sampling strategy for this study. Per the classification system by Orton (2008), completed beads are those which were ground to a circular shape, had the presence of use-wear, or were completed and broken with more than 50% remaining. Age estimates were drawn from direct radiocarbon dates, dated archaeological layers, or bracketing layers. The majority of the specimens were analyzed in-person (n=1148), under a low-power magnification, and photographed with a digital microscope. We recorded three metric variables (bead diameter, aperture diameter, and thickness) wherever possible. These are the most frequently reported, standardized characteristics in published literature.
Data collection	The majority of the specimens were analyzed in-person (n=1148), under a low-power magnification, and photographed with a digital microscope. We recorded three metric variables (bead diameter, aperture diameter, and thickness) wherever possible. Measurements of bead diameter and shell thickness were recorded with Toolway 70401 digital calipers, and typed into a digital archive. The record of digital microscope photos was later processed in the freeware program ImageJ to measure the bead aperture diameter. One of the collections (Enkapune ya Muto) could not be assessed in person, and instead measurements were collected from digital microscope photos that were taken by Dr. Phil Slater, under the supervision of Prof. Stanley Ambrose.
Timing	There are no time-dependent effects in this measurement process. Data were recorded between 2012 and 2020, as collections became available.
Data exclusions	No data were excluded from our analysis.
Non-participation	No beads declined to participate in our study.
Randomization	Randomization was not required as there are no covariates.

Reporting for specific materials, systems and methods

We require information from authors about some types of materials, experimental systems and methods used in many studies. Here, indicate whether each material, system or method listed is relevant to your study. If you are not sure if a list item applies to your research, read the appropriate section before selecting a response.

Materials & experimental systems

n/a	Involved in the study	
☒	☐	Antibodies
☒	☐	Eukaryotic cell lines
☐	☒	Palaeontology and archaeology
☒	☐	Animals and other organisms
☒	☐	Human research participants
☒	☐	Clinical data
☒	☐	Dual use research of concern

Methods

n/a	Involved in the study	
☒	☐	ChIP-seq
☒	☐	Flow cytometry
☒	☐	MRI-based neuroimaging

Palaeontology and Archaeology

Specimen provenance	No new excavation / fieldwork was conducted for this study. Data was collected from archaeological specimens that were previously excavated either in their respective storage locations or to the responsible person who had exported them on-loan. For the latter type of assemblages, I did not study them in their storage museums, nor did I deposit them in their storage museums. An artifact ID

can be found in Supplementary Table 1 that lists provenience information, where available. Permission was obtained directly from the access point, either the storage museum or the researcher. The only dataset which required a formal permit was the White Paintings Shelter site, housed at the National Museum and Monuments in Gaborone, Botswana. Permission was issued to Jennifer Miller from the Ministry of Environment, Wildlife and Tourism (Reference EWT8/36/4 XXVI (26)), from July 23, 2014 to October 15, 2014.

Specimen deposition	All specimens examined in-person were returned to their respective locations, either to the museum storage where accessed or to the responsible researcher who had exported them on-loan. Apollo 11 Cave assemblage was studied and deposited in the National Museum of Namibia, Windhoek. Border Cave assemblage studied and deposited in the McGregor Museum, Kimberley. Daumboy 3 Rockshelter assemblage studied while on-loan to M.Prendergast, collection housed in the National Museum of Tanzania, Dar es Salaam. Dikbosch Rockshelter assemblage studied and deposited in the McGregor Museum, Kimberley. Enkapune ya Muto assemblage studied via digital photographs provided by P.Slater, collection housed in National Museums of Kenya, Nairobi. Kakapel Rockshelter assemblage studied while on-loan to S. Goldstein (E.Ndiema and N.Boivin), collection housed in the National Museums of Kenya, Nairobi. Magubike and Mlambalasi Rockshelter assemblages studied while on-loan to P. Willoughby, collection housed in the National Museum of Tanzania, Dar es Salaam; Mumba Rockshelter assemblage studied while on-loan to M.Prendergast (also A.Mabulla and M.Domingo-Rodriguez), collection housed in the National Museum of Tanzania, Dar es Salaam. Nelson Bay Cave assemblage studied and deposited in the Iziko Museums of South Africa, Cape Town. White Paintings Shelter assemblage studied and deposited in the National Museum of Gaborone, with permission from the Ministry of Natural Resources and Tourism, Government of the Republic of Botswana, Ministry of Environment, Wildlife and Tourism. Wonderwerk Cave assemblage studied and deposited in the McGregor Museum, Kimberley.
Dating methods	No new dates are provided, none were required for this study.

☒ Tick this box to confirm that the raw and calibrated dates are available in the paper or in Supplementary Information.

Ethics oversight	No ethics oversight was required in this study.

Note that full information on the approval of the study protocol must also be provided in the manuscript.
